# Neurodegenerative Diseases: Epidemiology, Risk Factors, Classification, Pathophysiology, Clinical Features, Diagnosis, and Therapeutic Strategies

**DOI:** 10.1002/mco2.71090

**Published:** 2026-10-23

**Authors:** Zimeng He, Jingjing Zeng, Guoqing Wang, Yuxin Li, Feng Zhang

**Affiliations:** ^1^ Key Laboratory of Basic Pharmacology of Ministry of Education and Joint International Research Laboratory of Ethnomedicine of Ministry of Education and Key Laboratory of Basic Pharmacology of Guizhou Province and Laboratory Animal Center Zunyi Medical University Zunyi Guizhou China; ^2^ State Key Laboratory of Discovery and Utilization of Functional Components in Traditional Chinese Medicine Guizhou Medical University Guiyang Guizhou China

**Keywords:** Alzheimer's disease, amyotrophic lateral sclerosis, diagnosis, neurodegenerative diseases, Parkinson's disease, therapeutics

## Abstract

Neurodegenerative diseases are a growing cause of disability worldwide, particularly as populations age. Although these disorders differ in etiology and clinical presentation, advances in genetics, neuropathology, and biomarker research have identified shared disturbances in protein homeostasis, mitochondrial function, and immune regulation. These processes may contribute to selective neuronal vulnerability and neural circuit dysfunction. These findings are refining disease classification and diagnosis, yet effective disease‐modifying therapies remain limited. Disease‐specific abnormalities remain essential to clinical interpretation and treatment selection. Existing reviews often focus on individual disorders or isolated mechanisms, leaving the links between shared biology and disease‐specific phenotypes insufficiently clear. We synthesize evidence on the epidemiology, risk factors, classification, and pathophysiology of Alzheimer's disease, Parkinson's disease, amyotrophic lateral sclerosis, Huntington's disease, frontotemporal dementia, Friedreich's ataxia, and multiple sclerosis. We compare their clinical manifestations and diagnostic approaches, including the evolving role of biomarkers, and critically assess established and emerging therapies. By distinguishing shared mechanisms from disease‐specific abnormalities, we identify key translational gaps and explore how common biological disturbances may lead to distinct clinical trajectories. This cross‐disease perspective may inform diagnostic reasoning, treatment development, and future comparative research.

## Graphical abstract

Neurodegenerative diseases share convergent risk architectures and pathological mechanisms, yet diverge in selective vulnerability, clinical presentation, and therapeutic responsiveness. This review integrates cross‐disease epidemiology, pathophysiology, diagnosis, and treatment, highlighting the transition from symptomatic care toward biomarker‐guided and precision neurotherapeutic strategies.

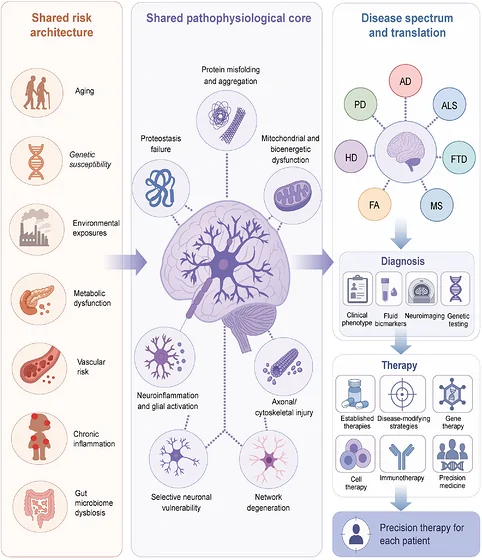

## 1 Introduction

Neurodegenerative diseases (NDDs) are clinically and biologically heterogeneous disorders characterized by progressive neuronal dysfunction and loss. Alzheimer's disease (AD), Parkinson's disease (PD), amyotrophic lateral sclerosis (ALS), Huntington's disease (HD), frontotemporal dementia (FTD), and Friedreich's ataxia (FRDA) have historically been classified as distinct clinicopathological entities based on their dominant clinical syndromes and characteristic pathology. Multiple sclerosis (MS) is classified primarily as an inflammatory demyelinating disease, although progressive neuroaxonal loss is central to its long‐term course [1, 2, 3, 4, 5, 6, 7]. This disease‐centered classification remains essential to clinical practice. However, advances in genetics and molecular neuropathology increasingly show that diagnostic boundaries do not fully reflect biological overlap among these disorders [8, 9, 10].

The public health burden of these disorders is increasing as populations age. Their prolonged courses cause cumulative disability and place growing demands on patients, families, and healthcare systems [11]. Over the past two decades, the field has moved from predominantly syndrome‐based descriptions toward biologically informed classification. Human genetics, molecular neuropathology, and fluid and imaging biomarkers have strengthened links among clinical phenotypes, underlying pathology, and potentially targetable biological processes [1, 12]. Emerging methods for detecting phosphorylated tau and nitrated α‐synuclein (α‐syn) illustrate progress in biomarker analysis, but they require rigorous clinical validation before routine use [13, 14]. Despite these advances, durable disease modification remains uncommon across many NDDs, highlighting the gap between mechanistic discoveries and clinical benefit [2, 3, 8].

Protein misfolding and aggregation, impaired proteostasis, mitochondrial stress, and maladaptive neuroinflammation can reinforce one another. Together, these processes contribute to synaptic dysfunction, circuit failure, and selective neuronal vulnerability in AD, PD, ALS, HD, and FTD [8, 9, 10]. These shared mechanisms support comparisons across disorders, but they do not make the disorders biologically equivalent. FRDA is characterized by frataxin deficiency, mitochondrial iron dysregulation, and metabolic stress. In MS, immune‐mediated demyelination coexists with progressive neuroaxonal injury [6, 7]. Many reviews focus on individual disorders or specific mechanisms, leaving the links among epidemiology, risk factors, biological mechanisms, diagnosis, and treatment insufficiently explored across disorders. A comparative synthesis is therefore needed to examine how shared disturbances contribute to distinct clinical phenotypes while accounting for differences in etiology, pathology, and treatment.

This review first summarizes the epidemiology and risk factors of the seven disorders, then compares their classification and pathophysiology. It next examines clinical manifestations and diagnostic approaches, including biomarker‐supported assessment and the challenge of distinguishing overlapping syndromes from disease‐specific patterns. Established and emerging therapies are then evaluated in light of their mechanistic rationale and the available preclinical and clinical evidence. Finally, we synthesize similarities and differences across disorders, identify major translational gaps, and outline priorities for future research. This sequence connects population‐level burden and risk with biological mechanisms, clinical assessment, and treatment while retaining disease‐specific distinctions.

## 2 Epidemiology and Risk Factors

The global burden of NDDs is increasing, although trends vary across disorders. Population aging contributes to this rise in many conditions. Genetic susceptibility, environmental exposures, lifestyle factors, and systemic metabolic and inflammatory processes shape risk to varying degrees. This section first summarizes shared epidemiological patterns across NDDs. It then examines disease‐specific epidemiological profiles and risk factors in AD, PD, ALS, HD, FTD, FRDA, and MS.

### 2.1 General Principles

NDDs pose a growing global health challenge, partly because populations are aging [15, 16, 17]. Despite differences in clinical presentation and patterns of neuronal vulnerability, older age is a major nonmodifiable risk factor for many NDDs [18].

Beyond age, genetic susceptibility, environmental exposures, and lifestyle factors may contribute to disease risk, although their relative importance varies by disorder [19]. Genome‐wide association studies (GWAS) have identified shared genetic associations among some NDDs, including loci related to lipid metabolism, neuroinflammation, and endolysosomal function [20]. Environmental neurotoxicants, particulate air pollution, and physical inactivity have also been examined as potential risk factors, with evidence varying by disorder [21, 22, 23].

Systemic processes may further influence disease susceptibility and progression. Chronic systemic inflammation, metabolic syndrome, and age‐related neuroendocrine changes may disrupt the blood–brain barrier (BBB) and promote neuroinflammation [24]. Gut microbiome dysbiosis has also been linked to several NDDs, potentially through systemic inflammation, microbial metabolites, and gut–brain signaling [25].

Shared susceptibility factors provide a basis for comparison, while disease‐specific genetics, exposures, and patterns of neural vulnerability help explain the distinct epidemiological profiles discussed below.

### 2.2 Disease‐Specific Features

#### 2.2.1 Alzheimer's Disease

AD is the leading cause of dementia in later life, accounting for approximately 60–70% of cases [26, 27]. Longer life expectancy and population aging contribute to the rising burden of AD, while improved detection affects reported estimates. Underdiagnosis remains common in low‐ and middle‐income countries because of limited healthcare resources and sociocultural barriers [28, 29, 30]. China, India, and the USA have large populations living with AD, and the burden in Japan is increasing. Reports from Africa, Latin America, and Southeast Asia also indicate rising prevalence, underscoring regional differences in the global burden of AD [31, 32, 33, 34, 35].

AD contributes to mortality and disability and increases the need for long‐term care. These effects place sustained pressure on families, caregivers, and healthcare systems [36]. Women have a higher lifetime risk of AD than men [37]. This difference may reflect longevity and unequal access to education, employment opportunities, and healthcare, as well as poverty and discrimination, particularly in low‐ and middle‐income countries [38]. Sex differences may also affect disease course. Some studies report faster neurodegeneration and cognitive decline in women and shorter survival after diagnosis in men [39].

AD is multifactorial. Some associated factors may be prodromal features or reflect reverse causation and therefore require cautious interpretation. Rare early‐onset familial AD is associated with pathogenic variants in *APP*, *PSEN1*, and *PSEN2* [40]. Late‐onset sporadic AD has a more complex etiology. The apolipoprotein E (*APOE*) ε4 allele remains the strongest established genetic risk factor for late‐onset AD [41, 42, 43]. GWAS also implicate lipid metabolism, immune responses, and endosomal and vesicular trafficking in AD susceptibility [44]. Dementia in older adults often reflects mixed pathology, including AD pathology, cerebral small‐vessel disease, and other age‐related changes. Risk factors identified in life‐course dementia frameworks should therefore not automatically be treated as AD‐specific causes with similar effect sizes [38]. Diabetes, hypertension, obesity, and dyslipidemia are potentially modifiable conditions associated with AD risk [45, 46, 47, 48, 49, 50, 51]. Potential mechanisms include reduced cerebral perfusion, BBB dysfunction, neurovascular imbalance, impaired insulin signaling, and chronic low‐grade inflammation. These processes may contribute to amyloid‐β (Aβ) accumulation, tau pathology, and neurodegeneration [52]. Midlife is therefore an important period for addressing hypertension, diabetes, and obesity.

Socioeconomic circumstances and behavior may also influence AD risk. Lower educational attainment is associated with higher AD risk in observational and genetic epidemiological studies. Higher education and cognitively stimulating activities have been linked to lower risk or later onset [53, 54, 55, 56, 57, 58, 59, 60, 61]. Lifelong cognitive engagement may support neural network efficiency and compensatory capacity. Reduced social contact, loneliness, depression, and chronic stress have also been associated with higher dementia risk [62, 63, 64]. These psychosocial associations do not necessarily establish a one‐way causal relationship, because some changes may be prodromal features of AD.

Several lifestyle exposures have been associated with lower AD risk, but observational findings alone do not establish protection. Observational studies associate the Mediterranean diet, Dietary Approaches to Stop Hypertension (DASH) diet, and Mediterranean–DASH Intervention for Neurodegenerative Delay diet with lower AD risk. Higher Healthy Eating Index 2015 scores show a similar association [65]. These findings suggest that overall dietary quality may be more informative than individual nutrients. Evidence that light‐to‐moderate alcohol consumption protects against AD is inconsistent and susceptible to confounding, survivor bias, and exposure misclassification. Recent imaging and genetic analyses have raised concerns about potential harm and found little evidence of a protective effect [66, 67, 68, 69]. Overall, these associations should be interpreted cautiously. Stronger longitudinal studies and appropriate intervention trials are needed to assess preventive strategies.

Risk reduction for AD should therefore be considered across the life course, with strategies tailored to individual risk profiles and multiple risk domains. Future studies should integrate biomarkers, pathological subtypes, and long‐term follow‐up to estimate the effects of risk modification and identify differences across populations.

#### 2.2.2 Parkinson's Disease

PD has become a major NDD with a steadily increasing global burden and has been described as the fastest‐growing neurological disorder worldwide [70]. More than 8.5 million people were living with PD in 2019. Disability‐adjusted life years and the number of PD‐related deaths increased markedly from 2000 to 2019, making PD an important public health challenge in aging societies. This burden reflects increasing incidence and prevalence, longer survival, and growing disability. It varies by age, sex, region, and diagnostic and management capacity [71].

Data from the Global Burden of Disease (GBD) 2021 study further show an overall increase in PD burden, with differences by age, sex, and region. China, in particular, has a high PD burden, with rising incidence and prevalence since 1990 and a higher burden in older adults and men. The increase in PD‐related deaths is largely attributable to population aging [72].

These estimates should be interpreted cautiously because GBD methodology, provincial data quality, screening awareness, and diagnostic overlap with atypical parkinsonian syndromes may affect them.

PD arises from the cumulative interaction of age‐related neural vulnerability, genetic susceptibility, and long‐term environmental exposures. Aging is the most important nonmodifiable risk factor, while genetic studies indicate that PD susceptibility includes both high‐risk variants and a polygenic component. *GBA1* and *LRRK2* genes are among the most important and common genetic risk factors for PD. However, most sporadic PD reflects interactions among aging, genetics, the environment, and host factors rather than a single determinant [73].

Among modifiable risk factors, environmental and occupational exposures are among the most studied [74]. Exposure to pesticides, particularly organochlorine pesticides, and occupational exposure in agriculture have been consistently associated with increased PD risk [74, 75]. Industrial solvents such as trichloroethylene, long‐term exposure to fine particulate matter, and traumatic brain injury have also been linked to PD risk. However, the strength of causal evidence and control of bias remain uncertain for some of these associations [74, 76, 77, 78]. Overall, these findings suggest that the rising PD burden may reflect not only aging but also chronic harmful exposures prevalent in industrialized societies.

Smoking and coffee intake show inverse associations with incident PD in epidemiological studies [79, 80, 81]. However, these findings should be interpreted as clues to disease mechanisms and differences in host response rather than as prevention recommendations, especially because the harms of smoking far outweigh any putative protective association.

PD risk factors should be distinguished from prodromal markers. Constipation, hyposmia, rapid eye movement sleep behavior disorder (RBD), and depression are associated with future PD development and are included in the Movement Disorder Society (MDS) research criteria for prodromal PD [82]. However, these symptoms may be early indicators of ongoing neurodegeneration rather than exogenous causal risk factors. Aging, genetic susceptibility, and environmental exposures should be considered separately when evaluating PD risk.

#### 2.2.3 Amyotrophic Lateral Sclerosis

The global epidemiological burden of ALS shows marked geographic heterogeneity, with substantial variation in reported incidence and prevalence across countries and regions. According to GBD estimates covering 1990–2016, the global all‐age annual incidence of motor neuron diseases was approximately 0.78 per 100,000 person‐years, and the prevalence was approximately 4.50 per 100,000 population. Incidence was generally higher in men and peaked at 70–79 years of age [83]. More recent registry studies indicate that the ALS burden is not static. A nationwide Danish study covering 1980–2021 reported increases in incidence and mortality, with a more pronounced burden among men and older adults [84]. Similarly, updated National ALS Registry estimates project that the number of people living with ALS in the USA will continue to increase through 2030 [85]. Together, these findings suggest that population aging and improved case ascertainment may increase the observed burden of ALS and the demand for medical and care services.

Like many NDDs, ALS is unlikely to result from a single cause. Instead, it more plausibly reflects long‐term interactions among age‐related vulnerability, genetic susceptibility, and environmental exposures. Approximately 85% of patients with ALS have no discernible family history and are typically classified as having sporadic ALS [86]. However, advances in genetic research have identified pathogenic or likely pathogenic variants in approximately 70% of familial ALS cases and 10–20% of sporadic ALS cases. *C9orf72*, *SOD1*, *TARDBP*, and *FUS* are among the major causative genes identified to date [87].

Among nongenetic risk factors, age, family history, and smoking are currently considered relatively robust, with smoking representing one of the few modifiable factors with clear public health relevance. A systematic review, meta‐analysis, and dose‐response analysis in 2024 supported a positive association between smoking and increased ALS risk, possibly more pronounced in women [88]. Other reported environmental and lifestyle factors include strenuous physical activity, head trauma, military service, pesticide exposure, lead exposure, electric shock, and farming occupations [89]. However, the evidence for many of these factors remains inconsistent. A 2025 umbrella review noted substantial heterogeneity across reported associations and possible residual confounding; therefore, most environmental risk factors cannot yet be regarded as fully established causal factors. Head trauma is a representative example: its reported association with ALS does not, by itself, establish causality [90].

Overall, ALS epidemiological research is moving beyond descriptions of incidence and prevalence toward a more integrated understanding of changing global burden, genetic stratification, and gene–environment interactions.

#### 2.2.4 Huntington's Disease

HD is a rare autosomal dominant NDD and the most common form of hereditary chorea. An updated systematic review and meta‐analysis of studies published between 2010 and 2022 estimated a global pooled incidence of approximately 0.48 cases per 100,000 person‐years and a global pooled prevalence of approximately 4.88 cases per 100,000 people. In subgroup analyses, incidence was higher in Europe and North America than in Asia, while prevalence was higher in Europe and North America than in Africa [91]. Variation in published prevalence estimates reflects not only population genetic background but also differences in case ascertainment, denominator definition, access to molecular testing, and inclusion criteria [92]. HD most commonly begins between 30 and 50 years of age, although juvenile‐ and childhood‐onset cases occur in only a small minority of patients [93].

The central determinant of HD is an inherited pathogenic CAG repeat expansion in the huntingtin (*HTT*) gene. Unlike most NDDs, HD is fundamentally a monogenic disorder rather than a disease primarily driven by environmental exposure. Clinically, 36–39 CAG repeats generally fall within the reduced‐penetrance range, whereas 40 or more repeats are usually fully penetrant. Larger expansions are associated with earlier onset [94]. However, inherited CAG repeat length alone cannot fully explain age at onset or variability between individuals. Recent studies have highlighted the roles of somatic CAG repeat expansion and DNA mismatch repair (MMR) mechanisms in modifying disease onset and progression.

A single‐cell sequencing study proposed the extra‐long repeats acquire toxic effect (ELongATE) model. It suggests that somatic CAG expansion in striatal neurons and its expansion rate, rather than inherited repeat length alone, may help determine disease onset [95]. In vivo clustered regularly interspaced short palindromic repeats (CRISPR) screening further identified DNA MMR genes, including *MSH2, MSH3, MLH1*, and *MLH3*, as enhancers of repeat expansion. It also identified *FAN1* and *PMS2* as suppressors [96]. The internal sequence context of the repeat tract is also clinically relevant. Loss of specific interruption sequences can advance disease onset and increase the likelihood of symptoms in individuals carrying reduced‐penetrance alleles [97]. Intergenerational instability also affects disease risk, with anticipation more common after paternal transmission [98].

Nongenetic factors are more commonly regarded as modifiers of age at onset or progression rather than primary determinants of HD development. Longitudinal studies in premanifest carriers suggest that smoking status, alcohol exposure, body mass index, educational attainment, and comorbidity burden may be associated with subsequent disease trajectories. However, these associations should be interpreted cautiously, as pathogenic *HTT* expansion remains the primary determinant of HD [99].

The epidemiology of HD is also shaped by inequities in case ascertainment. Diagnostic delays have been reported among Black patients in the USA and may be even longer in Latin America [100]. Enroll‐HD remains dominated by White participants, indicating substantial under‐representation of minority populations [101]. Such diagnostic delays may lead to an underestimation of true prevalence, while under‐representation in research may limit the generalizability of findings to diverse populations.

#### 2.2.5 Frontotemporal Dementia

FTD is an important cause of young‐onset dementia, but its epidemiological burden remains underestimated. A recent population‐based meta‐analysis reported a pooled crude incidence of 2.28 cases per 100,000 person‐years and a pooled crude prevalence of 9.17 cases per 100,000 people. Subtype‐specific incidence estimates were lower for behavioral variant FTD (bvFTD) and primary progressive aphasia (PPA) [102]. Age remains central to FTD epidemiology. Population‐based, age‐stratified data show that FTD also occurs after the age of 65 years and suggest that its incidence may increase with age [102, 103].

Epidemiological estimates for FTD vary substantially, partly because case ascertainment differs between PPA and bvFTD [102]. This heterogeneity is closely linked to clinical recognition bias. Early personality, behavioral, and psychiatric changes in bvFTD may be misdiagnosed as depression, bipolar disorder, or other primary psychiatric conditions. Such misclassification can delay diagnosis and distort incidence and prevalence estimates [104].

The most robust determinant of FTD risk is genetic susceptibility rather than broad lifestyle exposures. Approximately 30–50% of patients have a relevant family history, and around 10% show autosomal dominant inheritance. *C9orf72*, *GRN*, and *MAPT* are the most common causative genes [105, 106]. Regional variation in the frequencies of pathogenic variants in these genes indicates that FTD epidemiology is closely linked to population genetic background.

Evidence for nongenetic FTD risk factors remains limited, and a causal framework comparable to that for AD has yet to emerge. Reported associations with head trauma, thyroid disease, diabetes, and autoimmune disease are based mainly on small or retrospective studies. Because these studies are vulnerable to recall bias, differences in case definitions and confounding, the associations should be interpreted as suggestive rather than definitive [107, 108].

A multicenter study found lower educational attainment and more heart disease in sporadic FTD than in familial FTD, with the cardiovascular difference especially pronounced in bvFTD [106]. Familial FTD is mainly driven by highly penetrant genetic factors. Thus, modifiable factors may play a greater role in sporadic FTD than in genetic FTD. However, the evidence remains too exploratory to support risk stratification or prevention models comparable to those for AD.

#### 2.2.6 Friedreich's Ataxia

FRDA is a rare autosomal recessive NDD, but in certain ancestry groups it represents one of the most common early‐onset inherited ataxias. Epidemiological estimates are highly dependent on case ascertainment methods and population background. According to GeneReviews, prevalence estimates in populations predominantly of European ancestry differ according to whether diagnosis is based on clinical assessment alone or on both clinical and molecular testing. The estimated carrier frequency ranges from approximately one in 60 to one in 100 [109].

FRDA typically begins in childhood or adolescence, but age at onset varies widely. A 2025 review in *The Lancet Neurology* noted that FRDA usually begins during adolescence but may present from early childhood to late adulthood [110]. Similarly, GeneReviews reports onset ranging from before age 5 to after age 50. FRDA should therefore not be described solely as a childhood disease: it is typically early‐onset but also includes late‐onset and very‐late‐onset forms [109].

The geographic and ethnic distribution of FRDA is also markedly uneven. FRDA is more common in Europe, the Middle East, South Asia, and North Africa, but is exceedingly rare in China, Japan, Southeast Asia, and sub‐Saharan Africa [109, 111]. Thus, FRDA epidemiology is strongly shaped by ancestry and historical founder effects. Prevalence estimates, diagnostic rates, and genetic profiles may therefore not be directly comparable across regional cohorts.

FRDA is caused by biallelic pathogenic variants in the frataxin (*FXN*) gene. Most patients carry GAA repeat expansions on both alleles. A small minority carry a GAA expansion on one allele and a nonexpansion pathogenic variant on the other [112].

Among known determinants, the GAA repeat length of the shorter *FXN* allele (GAA1) is the most stable and informative predictor of phenotype. A longer GAA1 repeat is associated with earlier onset and more severe disease. However, GAA1 repeat length explains only part of the variance in age at onset [110].

A 2022 natural history study suggested that environmental factors and unidentified genetic modifiers may contribute substantially to variation in age at onset [113]. A 2024 study on modifier mechanisms in repeat expansion diseases further suggested that MMR‐related factors may influence repeat instability in FRDA, with potential effects on age at onset and disease progression [114]. However, this evidence concerns possible disease modifiers and does not establish exogenous risk factors for FRDA.

#### 2.2.7 Multiple Sclerosis

MS is one of the most important inflammatory diseases of the central nervous system (CNS) in young adults and a major cause of nontraumatic neurological disability. Its global burden has continued to rise, although estimates vary across data sources. The Atlas of MS estimated that the global number of people with MS had increased to approximately 3.1 million [115].

Geographically, MS shows marked regional heterogeneity. North America and Western Europe remain among the regions with the highest prevalence, incidence, and disability burden. Higher latitude is also one of its most consistent epidemiological features. MS typically begins between the ages of 20 and 40 years, with a clear female predominance. It is increasingly viewed as a chronic neuroimmune disease that extends across the life course rather than solely as a disease of young‐adult onset [116, 117].

Etiologically, MS is best explained by a multifactorial model in which environmental exposures act on a background of genetic susceptibility. Among known genetic variants, HLA‐DRB1*15:01 is considered the most important and consistently replicated common genetic risk factor [118]. However, genetic factors alone cannot explain disease onset, highlighting the crucial role of environmental exposures in disease pathogenesis.

Among established environmental factors, the evidence for Epstein–Barr virus (EBV) is the most compelling. Longitudinal studies show that EBV infection precedes MS onset and is strongly associated with future MS risk [119, 120]. Smoking, low sunlight exposure or vitamin D insufficiency, and childhood or adolescent obesity may amplify risk in susceptible individuals. Evidence supports interactions between smoking and HLA‐related genetic backgrounds. Higher 25‐hydroxyvitamin D levels are associated with lower MS risk, whereas childhood or adolescent obesity is associated with increased risk [121, 122, 123].

Overall, the epidemiological and risk‐factor landscape of NDDs is best understood as a continuum rather than a single model. At one end of this continuum are disorders with a substantial, theoretically preventable burden, such as AD‐dominant late‐life dementia. PD, ALS, FTD, and MS are shaped by complex interactions among aging, environmental exposures, and genetic susceptibility. At the other end are HD and FRDA, in which pathogenic genetic variation overwhelmingly determines disease occurrence (Figure 1).

**FIGURE 1:**
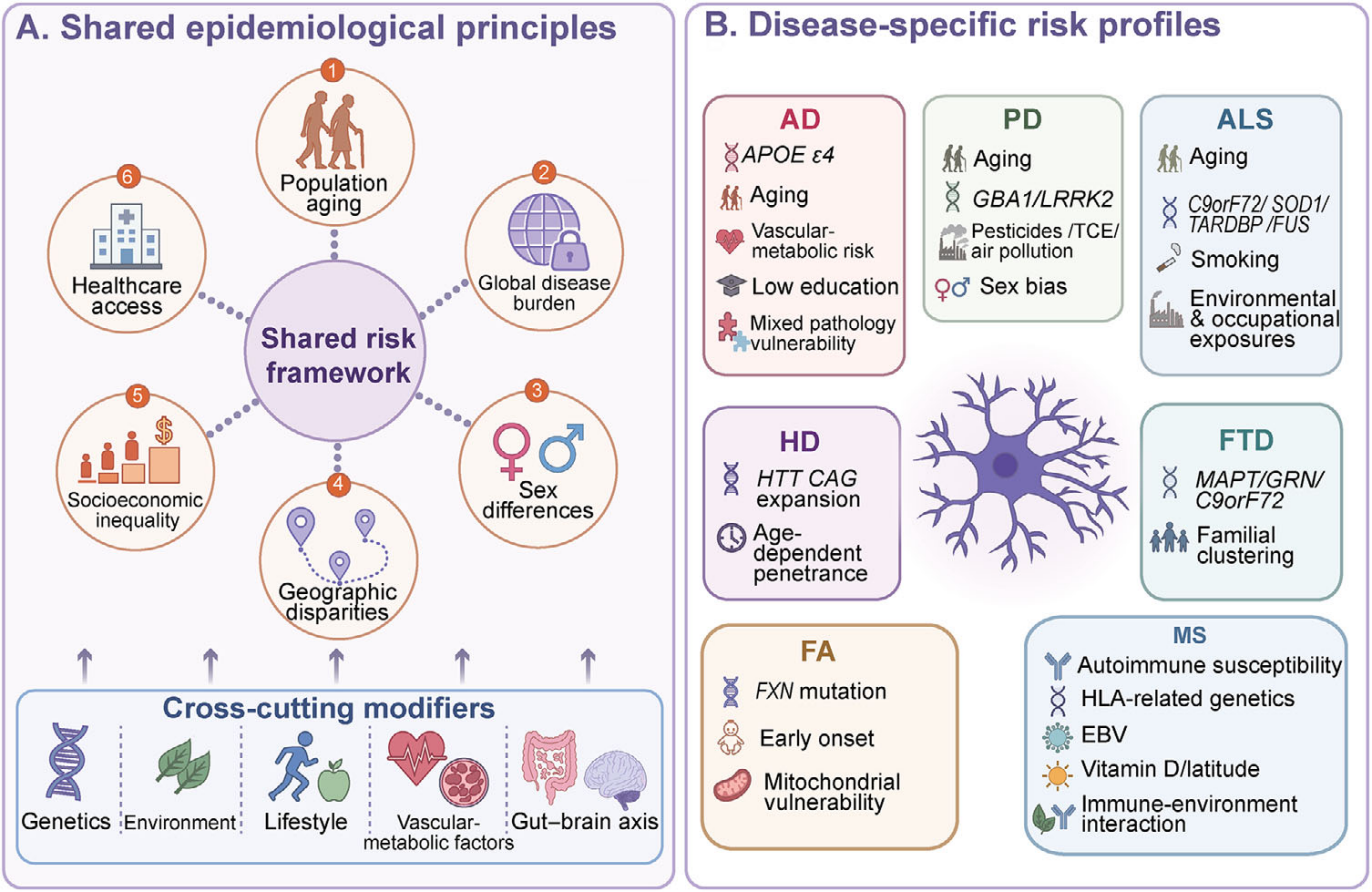
Cross‐disease epidemiology and risk architecture of NDDs. (A) Major NDDs share a common epidemiological framework characterized by aging as the dominant risk factor, rising global burden, sex‐related differences, and regional and socioeconomic disparities. These overarching patterns are shaped by cross‐cutting risk domains, including genetic susceptibility, environmental exposure, vascular and metabolic dysfunction, immune‐inflammatory imbalance, and gut–brain axis perturbation. (B) Despite these shared determinants, individual disorders retain distinct risk fingerprints. AD is strongly associated with APOE ε4 and vascular‐metabolic burden; PD with GBA1/LRRK2 variants and environmental toxins; ALS with major ALS‐related genes and exposure‐related risk; HD with HTT CAG expansion; FTD with MAPT, GRN, and C9orf72‐related susceptibility; FA with FXN mutation and early mitochondrial vulnerability; and MS with immune‐genetic susceptibility and environmental modifiers such as Epstein–Barr virus exposure and vitamin D deficiency. *Abbreviations*: AD, Alzheimer's disease; ALS, amyotrophic lateral sclerosis; FA, Friedreich's ataxia; FTD, frontotemporal dementia; HD, Huntington's disease; MS, multiple sclerosis; NDDs, neurodegenerative diseases; PD, Parkinson's disease.

## 3 Classification and Pathophysiology

The classification of NDDs is shifting from conventional syndrome‐based categories toward a biologically grounded framework centered on pathological hallmarks and molecular mechanisms. Although the dominant aberrant proteins and vulnerable neuroanatomical regions differ across disorders, NDDs converge on interlinked mechanisms. These include protein misfolding and propagation, proteostasis failure, selective vulnerability, network‐based degeneration, neuroinflammation, and glial dysfunction, as well as bioenergetic dysfunction, cytoskeletal disruption, and genomic damage. This section first introduces a pathology‐based classification framework, then examines mechanisms shared across diseases, and finally outlines disease‐specific classifications and pathophysiological features.

### 3.1 Pathology‐Based Classification Framework of NDDs

Compared with traditional syndrome‐based descriptions, a framework based on pathological hallmarks better integrates shared mechanisms of NDDs with disease‐specific differences. Traditional classifications based on dominant clinical phenotypes, such as cognitive impairment, movement disorders, behavioral changes, or ataxia, remain clinically useful. However, these classifications are increasingly limited by phenotypic overlap and within‐disease heterogeneity. The frequent codeposition of abnormal proteins, including tau, Aβ, α‐syn, and TDP‐43, in ageing brains or at advanced disease stages further complicates classification. Accordingly, the classification of NDDs has increasingly shifted from symptom‐based categories toward frameworks based on pathology and molecular features, with protein‐based taxonomies gaining importance in neuropathological diagnosis and mechanistic research [124].

This framework also has stronger explanatory value because many classical NDDs share proteinopathy‐related processes involving abnormal protein misfolding, aggregation, propagation, and impaired clearance. Despite differences in initiating molecules and affected systems, NDDs share several pathological features. These include protein aggregation, synaptic and network dysfunction, proteostasis failure, cytoskeletal abnormalities, impaired energy homeostasis, DNA/RNA dysregulation, inflammation, and neuronal death [8, 124].

At the disease‐specific level, this framework accommodates most disorders covered in this review. AD is characterized primarily by dual Aβ and tau pathology [125], and PD by α‐syn‐related synucleinopathy [126]. ALS and a subset of FTD are associated with TDP‐43 proteinopathy [127, 128], whereas HD involves polyglutamine expansion in mutant huntingtin and its downstream aggregation toxicity [129]. Thus, although the dominant abnormal proteins differ across diseases, each disorder can be understood within a shared pathological sequence linking aberrant proteins, cellular homeostatic failure, selective vulnerability, and neural network degeneration.

However, a classification based on pathological hallmarks should also accommodate disorders that do not fit classical proteinopathy models. FRDA is not primarily driven by classical abnormal protein aggregation but by impaired iron–sulfur cluster biogenesis, mitochondrial dysfunction, and metabolic imbalance caused by frataxin deficiency. It is therefore better understood as an NDD dominated by mitochondrial and metabolic injury [130]. MS cannot be fully captured by traditional relapsing‐remitting, secondary progressive, and primary progressive categories. Its histopathological core involves immune‐mediated demyelination, plaque formation, glial activation, chronic active lesions, and progressive neurodegeneration [131]. Therefore, this review adopts a classification based on pathological hallmarks while accounting for disorders with distinct dominant mechanisms.

### 3.2 Shared Pathophysiological Principles Across NDDs

Although NDDs differ in their initiating molecules, regions affected early in disease and clinical phenotypes, they are not biologically isolated entities. Instead, they converge on interconnected mechanisms, including abnormal protein aggregation, proteostasis failure, network dysfunction, selective vulnerability, inflammation, and impaired energy metabolism. Together, these processes can contribute to neuronal death.

#### 3.2.1 From Protein Misfolding to Propagative Neurotoxicity

In most NDDs, abnormal protein pathology serves both as a hallmark for disease classification and as a dynamic driver of tissue injury. Rather than merely forming visible deposits, abnormal proteins may progress through misfolding, toxic oligomerization, aggregate formation, and pathological spread. These processes can amplify damage from molecular changes and cellular injury to network‐ and systems‐level degeneration.

Mechanistically, protein misfolding reflects the conversion of proteins from relatively soluble and clearance‐competent states into more toxic, abnormal conformations. These aberrant conformations can disrupt proteostasis, synaptic function, axonal transport, mitochondrial activity, and organelle homeostasis. They can also trigger stress responses and cause cellular injury. As disease progresses, some abnormal proteins exhibit seed‐dependent amplification and prion‐like propagation. These processes allow focal pathology to spread along anatomical or functional networks and contribute to broader circuit damage.

Although different diseases share this general pattern, they differ in their dominant protein species, toxic conformations, vulnerable cell types, and modes of pathological spread. These differences shape their distinct pathological spectra and clinical phenotypes [8]. Thus, abnormal protein misfolding, aggregation, and propagation can be understood as shared mechanisms of pathological amplification that take distinct forms across diseases.

#### 3.2.2 Proteostasis Failure and Impaired Clearance

If protein misfolding and aggregation explain how toxic burden arises in NDDs, proteostasis failure and impaired clearance explain why this burden continues to accumulate. Under physiological conditions, cells maintain protein synthesis, folding, trafficking, remodeling, and degradation through molecular chaperones, the ubiquitin–proteasome system (UPS) and the autophagy–lysosome pathway (ALP). In NDDs, the problem is not merely the emergence of abnormal proteins, but the progressive loss of quality‐control capacity to handle misfolded proteins and damaged organelles. Thus, proteostasis failure can result from aggregation and, in turn, increase the pathological burden [8, 132].

Maintaining proteostasis is particularly important in neurons, which are long‐lived, terminally differentiated cells that cannot dilute damaged proteins or ageing organelles through cell division. Neurons therefore depend heavily on sustained quality‐control systems, especially the ALP. This pathway clears aggregation‐prone proteins, damaged mitochondria, synapse‐associated components, and other long‐lived cargo. When basal autophagy declines or clearance flux is blocked, the postmitotic state and complex morphology of neurons make them especially vulnerable to proteostasis failure. This helps explain why impaired clearance is a recurring feature across major NDDs [133].

Mechanistically, proteostasis failure reflects dysfunction at multiple levels of the quality‐control system rather than at a single step. Defects may involve reduced chaperone capacity, impaired recognition of misfolded proteins, and reduced UPS efficiency. Within the ALP, they may disrupt autophagosome maturation, lysosomal fusion, and downstream lysosomal function. Ageing further reduces ALP efficiency, making related defects more likely to manifest in AD, PD, and FTD [134, 135].

Beyond allowing cellular waste to accumulate, impaired clearance can reshape the cellular environment and amplify protein toxicity. Lysosomal acidification illustrates this relationship because acidification mediated by vacuolar‐type H+‐ATPase (V‐ATPase‐mediated acidification) supports autophagic degradation, mitochondrial quality control, and interorganelle communication. When acidification is impaired, defective degradation can contribute to broader endomembrane imbalance, energetic stress, and amplified cellular stress responses [136].

#### 3.2.3 Selective Vulnerability and Network‐Based Degeneration

Although NDDs share several core pathological mechanisms, tissue injury does not occur uniformly. Different diseases preferentially affect specific brain regions, cell types, and functional systems, a phenomenon known as selective vulnerability. Under similar conditions of ageing, inflammation, proteotoxicity, or metabolic stress, neurons and glial cells differ in their tolerance to injury. Consequently, early pathology often emerges in highly vulnerable nodes [137].

At the cellular level, selective vulnerability reflects both cell‐autonomous and noncell‐autonomous mechanisms. Different cell populations vary in metabolic burden, axonal length, firing activity, calcium homeostasis, protein quality‐control capacity, and stress‐response programs. These differences help determine which cells fail first under pathological stress [137]. Astrocytes also shape vulnerability or resilience through metabolic support, synaptic regulation, and inflammatory amplification [138]. Thus, selective vulnerability should be understood as an interaction between intrinsic cellular features and the local microenvironment, rather than as a simple regional phenomenon.

In NDDs, pathology extends beyond vulnerable cells and involves network‐level spread and reorganization. Connectome studies suggest that neurodegeneration often follows predictable trajectories along pre‐existing structural and functional connections [139]. Brain networks may serve as conduits for pathological proteins and aberrant activity. Their topological properties can influence how pathological burden spreads between regions and shape progression trajectories [140].

Network‐level degeneration can therefore be viewed as an amplification of selective vulnerability. From a connectomic perspective, pathology preferentially targets highly connected, metabolically demanding hubs, including rich‐club regions involved in extensive information integration [141]. Selective degeneration of these core nodes has been observed in multiple diseases, including AD and HD [142, 143]. Their loss may promote broader functional disconnection across neighboring circuits and long‐range connections.

#### 3.2.4 Neuroinflammation and Glial Dysfunction

Neuroinflammation and glial dysfunction are shared pathogenic processes in NDDs. They involve remodeling of CNS immune homeostasis, glial support functions, and the microenvironment supporting neuronal survival, rather than merely increased inflammatory mediator levels [8, 144]. Microglia mediate immune surveillance, synaptic pruning, and injury responses. Astrocytes maintain ionic homeostasis, neurotransmitter clearance, metabolic support, and BBB‐related functions. Oligodendrocytes support axonal conduction through myelination and metabolic coupling. When these functions become chronically dysregulated, glial cells shift from responders to active participants in disease progression [144, 145, 146].

Single‐cell and single‐nucleus transcriptomic studies show that glial responses in NDDs cannot be reduced to M1/M2 or resting/activated dichotomies. Instead, they comprise heterogeneous reactive states that vary across space and time, including disease‐associated microglia and reactive astrocyte subtypes [147, 148, 149]. These findings support a continuum model of glial responses, in which early activation may be compensatory, promoting phagocytosis and debris clearance while limiting local injury. However, persistent stimulation can shift glia toward chronic neurotoxic states marked by inflammatory mediator release, synaptic disruption, and heightened neuronal vulnerability [144].

The triggering receptor expressed on myeloid cells 2 (TREM2)–APOE signaling axis can shift microglia from a homeostatic state toward a neurodegeneration‐associated phenotype, with altered programs for phagocytosis, lipid handling, and immune modulation [150]. This suggests that microglia undergo functional reprogramming in the disease environment, potentially altering their clearance of abnormal proteins and responses to neurons, myelin, and synaptic structures. Astrocyte dysfunction is also a key component of shared pathophysiology. Inflammatory signals released by activated microglia can induce neurotoxic reactive astrocytes. These astrocytes lose neurotrophic and homeostatic functions and can directly damage neurons and oligodendrocytes [151].

The roles of oligodendrocytes and their precursor cells in the progression of NDDs extend beyond traditional white‐matter disorders. Impaired myelin homeostasis weakens axonal conduction and metabolic support, exacerbating axonal stress, lipid peroxidation, and local inflammation. These effects link glial dysfunction to neuronal degeneration [152]. Together, these mechanisms support the view that neuroinflammation is part of glia–neuron unit dysfunction rather than an isolated immune phenomenon.

#### 3.2.5 Bioenergetic, Cytoskeletal, and Genomic Dysfunction

Beyond proteostasis and glial responses, bioenergetic dysfunction, cytoskeletal imbalance, and genomic damage constitute another shared pathological axis in NDDs. These abnormalities are tightly coupled in terminally differentiated neurons with high metabolic demands and long axons, shaping cellular tolerance to chronic stress.

Bioenergetic dysfunction occurs across multiple NDDs. Neurons depend heavily on adenosine triphosphate (ATP) supply, calcium homeostasis, and redox balance. Persistent mitochondrial injury can therefore reduce ATP production, increase reactive oxygen species (ROS) burden, impair calcium buffering, and activate cell‐death pathways. These changes can directly impair synaptic transmission, axonal maintenance, and neuronal plasticity, thereby amplifying protein aggregation and inflammatory responses [153, 154].

Cytoskeletal disruption and axonal transport impairment also render neurons vulnerable to degeneration. Neurons rely on microtubules, actin, and motor proteins to transport mitochondria, vesicles, lysosomes, and RNA complexes between the soma and distal synapses. When cytoskeletal stability declines, early consequences include distal synaptic dysfunction, impaired axonal trophic support, and local energy imbalances. Axonal transport impairment is therefore regarded as an early and unifying event in neurodegeneration. It can be triggered by tau abnormalities, α‐syn toxicity, or motor protein dysfunction and can further promote abnormal protein accumulation and axonal degeneration [155].

Genomic instability and defective DNA repair are also important shared mechanisms in NDDs. Long‐lived, postmitotic neurons are particularly vulnerable to the accumulation of DNA damage because of their limited renewal capacity and sustained metabolic and oxidative stress (OS). Dysregulated DNA damage responses or repair programs can lead to transcriptional abnormalities, disrupted chromatin homeostasis, synaptic dysfunction, and cell death [156]. Within the ALS–FTD spectrum, dysfunction of RNA/DNA‐binding proteins such as TDP‐43 and FUS is also closely linked to impaired DNA repair [157].

### 3.3 Disease‐Specific Features

#### 3.3.1 Alzheimer's Disease

The classification of AD has shifted from a purely clinical syndrome‐based framework toward a biologically grounded continuum. Traditional staging describes progression from a preclinical phase to mild cognitive impairment (MCI) and dementia. This sequence was formalized in the 2011 National Institute on Aging–Alzheimer's Association (NIA‐AA) guidelines [158, 159]. Recommendations for preclinical AD specifically addressed individuals without overt symptoms [160]. With advances in biomarker research, the AT(N) framework groups biomarkers of Aβ deposition (A), tau pathology (T), and neurodegeneration (N). This allows AD‐related pathology to be characterized before clinical symptoms appear [161]. Proteomic and systems biology studies further reveal molecular subtypes involving synaptic plasticity, innate immune activation, RNA regulation, choroid plexus dysfunction, and BBB injury. These findings support a view of AD as a biologically heterogeneous continuum rather than a single uniform entity [162].

Mechanistically, AD is better understood as a network of interacting pathological processes rather than a single linear cascade. The amyloid cascade hypothesis proposes that Aβ deposition resulting from abnormal processing of APP initiates downstream neurodegenerative changes [163]. Familial AD caused by variants in *APP*, *PSEN1*, and *PSEN2* supports the role of excessive Aβ production and aggregation [164, 165, 166, 167]. However, Aβ alone is insufficient to explain disease progression, whereas tau pathology is more closely associated with neuronal dysfunction and clinical severity [168, 169]. Under pathological conditions, hyperphosphorylated tau detaches from microtubules, destabilizes the cytoskeleton, and impairs axonal transport. It also aggregates into neurofibrillary tangles, contributing to neuronal degeneration and cognitive impairment [170, 171, 172]. Aβ and tau also interact synergistically. Aβ can promote tau phosphorylation, aggregation, and propagation. Tau, in turn, can amplify Aβ‐induced circuit abnormalities and synaptic injury, thereby promoting tau spread and network dysfunction [173, 174, 175].

Beyond Aβ and tau, AD involves interactions across glial, metabolic, and synaptic domains. Astrocyte activation may link tau pathology with neurodegenerative injury [176]. Chronic neuroinflammation, mediated by microglia and astrocytes, can amplify amyloid and tau pathology and further promote neurodegeneration [177]. Microglial responses vary with disease stage and cell state. Early activation may facilitate Aβ clearance, whereas persistent activation can impair phagocytosis, increase proinflammatory cytokine release, and aggravate neuronal injury and synaptic loss [178, 179]. Mitochondrial dysfunction and metabolic imbalance form another key axis, as disrupted mitochondrial dynamics, impaired ATP synthesis and OS threaten energy‐demanding neurons [180]. Synaptic dysfunction includes reduced plasticity, disrupted neurotransmitter signaling, and progressive synapse loss. It reflects the combined effects of upstream Aβ toxicity, tau‐mediated cytoskeletal disruption, mitochondrial dysfunction, and neuroinflammation, and constitutes a common final pathway driving cognitive decline [181].

#### 3.3.2 Parkinson's Disease

PD classification has traditionally been based on clinical features, including age at onset, motor phenotype, and nonmotor symptom burden. These categories include early‐ versus late‐onset PD, tremor‐dominant versus postural instability/gait difficulty‐dominant phenotypes, and subtypes with prominent cognitive or psychiatric features. They remain useful for describing heterogeneity, prognosis, and treatment response [182, 183]. Such classifications partly reflect interpatient differences in symptom spectrum and progression rate, supporting their use in clinical research and preliminary prognostic assessments.

However, clinical subtyping alone has clear limitations. Systematic reviews by the MDS and recent analyses of early PD subtyping have identified substantial methodological heterogeneity, limited reproducibility, and insufficient biological grounding in phenotypic and data‐driven classifications [182, 184].

Accordingly, PD classification is shifting toward biological stratification. The 2024 SynNeurGe research diagnostic criteria incorporate evidence of pathological α‐syn in tissues or cerebrospinal fluid (CSF), neuroimaging evidence of neurodegeneration, and pathogenic gene variants [185]. They link these biological components with clinical features. The MDS statement further emphasizes that biological definition, staging, and classification are conceptually distinct. It positions current biological frameworks mainly as tools for research stratification and mechanistic investigation, rather than replacements for routine clinical diagnosis [186].

The pathophysiological basis of PD reflects interactions among proteostasis failure, organelle dysfunction and neuroimmune responses. α‐syn misfolding, aggregation and propagation are central to PD pathogenesis. The identification of α‐syn as a major component of Lewy bodies established the basis for defining PD as a synucleinopathy [187]. Experimental and staging studies further suggest that α‐syn pathology can propagate along neural networks and contribute to synaptic dysfunction and neuronal death [188, 189]. Thus, abnormal α‐syn is both a pathological hallmark and a mechanism driving disease progression.

Mitochondrial dysfunction represents another key axis of PD pathophysiology. Nigral dopaminergic neurons are highly sensitive to metabolic stress and OS. Disruption of the PTEN‐induced kinase 1 (PINK1)/Parkin pathway can impair recognition of damaged mitochondria, mitophagy and mitochondrial quality control [190]. This links PD‐related neuronal injury to early disruption of mitochondrial surveillance, repair and clearance rather than solely to terminal cell death.

ALP dysfunction also bridges genetic susceptibility and sporadic pathology. Reduced activity of glucocerebrosidase (GCase), encoded by *GBA1*, can promote α‐syn aggregation. Accumulated α‐syn can further inhibit GCase function, forming a self‐amplifying loop between impaired degradation and abnormal protein accumulation [191]. Together, these mechanisms inform therapeutic strategies targeting *GBA1*, *LRRK2*, and lysosomal homeostasis.

Neuroinflammation and immune responses also contribute to PD pathophysiology. Microglial activation has been observed in the substantia nigra and more broadly in the brains of patients with PD, even at relatively early stages [192, 193]. Adaptive immunity may also participate in PD. T cells from patients with PD can recognize α‐syn‐derived antigenic peptides, linking abnormal protein aggregation to peripheral immune recognition and inflammatory amplification [194].

Gut–brain axis studies further suggest that gut microbiota may modulate α‐syn pathology, neuroinflammation, and motor phenotypes [195].

#### 3.3.3 Amyotrophic Lateral Sclerosis

ALS classification has traditionally been based on clinical phenotypes, including site of onset, upper and lower motor neuron involvement, and patterns of disease progression. Limb‐onset ALS is the most frequent form, whereas bulbar‐onset ALS is generally associated with faster functional decline and poorer prognosis [196]. Several motor phenotypes are clinically recognized and differ in symptom distribution, progression rate, and survival outcomes [197, 198].

However, classifications based solely on onset site or classical phenotype labels have limitations, including variation in subtype nomenclature, unclear boundaries between subtypes and inconsistent classification of rare phenotypes. The 2025 expert consensus therefore proposed the OPM classification to improve consistency in clinical communication and research stratification. This three‐dimensional framework uses the region of onset (O), propagation of motor symptoms (P), and degree of upper and/or lower motor neuron dysfunction (M) [197].

Beyond clinical phenotypes, genetic stratification is an important dimension of ALS classification. ALS is a heterogeneous syndrome involving multiple genes, including *C9orf72*, *SOD1*, *FUS*, and *TARDBP*. Different genotypes may be associated with differences in age at onset, clinical phenotype, cognitive‐behavioral involvement, and disease progression. For example, *SOD1*‐related ALS has a specific molecular pathological basis. *C9orf72*‐related cases are often associated with a higher risk of frontotemporal dysfunction [199, 200]. Molecular subtyping offers another approach to ALS classification. Transcriptomic studies suggest that ALS can be stratified into molecular subtypes associated with glial activation, OS, or aberrant transposon activation. These subtypes may correlate with clinical features and survival differences [201]. However, molecular subtyping remains mainly a research tool. Its reproducibility, interpretability, and clinical feasibility remain insufficient for routine clinical classification.

ALS pathophysiology arises from interactions among RNA metabolic dysregulation, aberrant protein aggregation, organelle dysfunction, impaired axonal transport, and neuroinflammation. TDP‐43 proteinopathy is one of the central pathological features of ALS. In most cases, TDP‐43 is mislocalized from the nucleus to the cytoplasm, where it forms abnormal aggregates. This mislocalization and aggregation can produce both toxic gain of function and loss of normal nuclear RNA‐processing functions [202, 203].

TDP‐43 pathology also highlights the central role of RNA metabolic dysfunction in ALS. RNA‐binding proteins such as TDP‐43 and FUS participate in splicing, posttranscriptional modification, RNA transport, and stress granule regulation. Their dysfunction can disrupt neuronal RNA homeostasis. TDP‐43 dysfunction can further cause aberrant polyadenylation, cryptic exon inclusion, and posttranscriptional dysregulation. These changes can affect motor neuron survival and help explain the ALS–FTD molecular continuum [204, 205].

ALS caused by a *C9orf72* hexanucleotide repeat expansion further highlights the importance of RNA pathology in this disease. This expansion can produce toxic gain of function through repeat RNA toxicity and dipeptide repeat proteins (DPRs). Loss of normal *C9orf72* function can impair autophagy, stress granule regulation, and intracellular trafficking. Together, these processes promote neurodegeneration [206]. Beyond RNA pathology, proteostasis failure and organelle dysfunction are also central to ALS pathophysiology. Misfolded protein accumulation may reflect impaired degradation via the UPS and the ALP, while mitochondrial dysfunction and OS contribute to motor neuron vulnerability. Abnormal proteins such as TDP‐43 and SOD1, together with glutamate excitotoxicity, can disrupt mitochondrial dynamics, energy metabolism, and calcium homeostasis. These disturbances can promote calcium overload, ROS generation, mitochondrial damage, and neuronal death [207]. These mechanisms form a self‐reinforcing cycle in which proteotoxicity aggravates organelle injury, which in turn promotes protein misfolding and aggregation. ALS pathophysiology also extends beyond motor neuron‐intrinsic mechanisms to noncell‐autonomous processes involving microglia, astrocytes, oligodendrocytes, and the peripheral immune system. These glial and immune cells contribute to motor neuron injury through the release of inflammatory mediators, impaired metabolic support, and changes in the synaptic environment. In addition, TDP‐43 aggregation, mitochondrial DNA release, and neuronal damage‐associated molecular patterns can activate innate immune pathways and amplify neuroinflammation [203].

#### 3.3.4 Huntington's Disease

HD classification is multidimensional, involving genetic penetrance, age at onset, and disease‐continuum staging. Genetically, CAG repeat length in the *HTT* gene provides the fundamental basis for classification. Alleles with 27–35 repeats are intermediate. Those with 36–39 repeats are pathogenic with reduced penetrance, whereas those with ≥40 repeats are generally fully penetrant pathogenic expansions [94]. This stratification informs genetic counseling and research inclusion criteria. In the HD Integrated Staging System (HD‐ISS), ≥40 repeats serve as the biological entry criterion. A repeat length of 36–39 alone does not meet this entry criterion [94, 208]. By age at onset, HD is predominantly an adult‐onset disorder. Juvenile‐onset HD usually refers to symptom onset before the age of 20 years and may feature bradykinesia, hypertonia, rigidity, seizures, and less prominent chorea [94, 209].

From a staging perspective, traditional HD frameworks distinguish premanifest and manifest disease, with manifest HD often divided into early, middle, and late stages [94]. By contrast, the HD‐ISS conceptualizes HD as a continuum from pathogenic expansion to measurable pathobiological changes, clinical phenotype, and functional decline. It divides this continuum into stages 0–3 [208]. This framework formally includes individuals traditionally considered presymptomatic and is useful for prodromal research and stratification in disease‐modifying trials. Shoulson–Fahn staging, based on the Total Functional Capacity scale, remains practical for manifest HD because it reflects declining independence in daily life [210].

HD pathophysiology originates from a CAG repeat expansion in exon 1 of the *HTT* gene, which produces mutant HTT. HD involves both toxic gain‐of‐function effects of mutant HTT and disruption of normal HTT function. Together, these changes lead to transcriptional dysregulation, mitochondrial dysfunction, proteostasis failure, impaired nucleocytoplasmic transport, and synaptic dysfunction. The resulting disturbances can reinforce one another during disease progression [211]. A major recent advance is the recognition that somatic CAG repeat expansion and DNA repair pathways modify HD onset and progression. Inherited repeat length alone does not determine disease onset and progression. Ongoing somatic expansion within vulnerable cells may convert genetic risk into local neurotoxicity. Consistent with this, modifier genes involved in MMR and other DNA repair pathways influence age at onset and disease trajectory. Measures of somatic expansion have also been associated with subsequent caudate and putamen atrophy [212, 213].

At the tissue and circuit levels, selective vulnerability of striatal medium spiny neurons remains central, followed by progressive involvement of the cortex and other brain regions [211]. Recent cell‐type‐specific studies suggest that somatic expansion is important for neuronal injury but may not, by itself, explain cell death. The vulnerability of corticostriatal projection neurons further indicates that HD involves degeneration across circuits beyond the striatum [214, 215]. Within cells, mutant HTT disrupts interconnected homeostatic pathways, including transcriptional regulation, neurotrophic support, synaptic plasticity, stress responses, ALP‐mediated clearance, mitochondrial function, and energy metabolism [211, 216]. Single‐nucleus transcriptomic studies further suggest that medium spiny neurons in prodromal HD already show glutamate excitotoxicity‐related signatures, indicating that synaptic abnormalities may precede overt cell death [217].

Non‐neuronal cells also contribute to HD progression. Astrocyte abnormalities in morphology, extracellular ion and neurotransmitter homeostasis, and metabolic support can amplify synaptic and energetic imbalances in the striatum. Microglial activation and peripheral immune‐related inflammation may also drive neuronal stress and disease progression [218, 219]. Thus, current understanding of HD integrates mutant protein aggregation with somatic expansion, cell‐type‐specific vulnerability, and interactions between intraneuronal and extraneuronal processes.

#### 3.3.5 Frontotemporal Dementia

FTD encompasses a spectrum of NDDs characterized by selective degeneration of the frontal and temporal lobes and by variation in clinical presentation, genetic background, and molecular pathology [5]. Its distinctiveness lies in relatively early onset, prominent vulnerability of frontotemporal networks and the lack of a simple one‐to‐one correspondence between clinical syndromes and molecular pathology [220, 221]. Pathologically, FTD commonly reflects underlying frontotemporal lobar degeneration (FTLD), which is classified primarily by its abnormal protein aggregates and associated cellular pathology [222].

The diversity of underlying protein pathology is central to FTD classification. The major FTLD subtypes include FTLD‐tau, FTLD‐TDP, and the less prevalent FTLD‐FUS, with FTLD‐tau and FTLD‐TDP accounting for most cases [222, 223, 224]. This framework uses the dominant deposited protein to subtype FTLD, even when clinical syndromes overlap. FTLD‐TDP can be further subdivided according to inclusion morphology and regional brain distribution. These subtypes illustrate how different molecular trajectories can converge in frontotemporal networks [223, 225].

Another defining feature of FTD is the close correspondence between genetic drivers and molecular pathology. The major causative genes include *MAPT*, *GRN*, and *C9orf72*, which account for a substantial proportion of familial FTD and aid in defining pathological subtypes. *MAPT* mutations are closely associated with tau pathology. *GRN* haploinsufficiency is linked to TDP‐43 pathology, lysosomal dysfunction, and disrupted glial homeostasis. *C9orf72* hexanucleotide repeat expansion links FTD and ALS through repeat RNA toxicity, DPR toxicity, and nucleocytoplasmic transport defects [222, 226, 227].

The ALS–FTD continuum further explains the distinctive position of FTD in NDD classification. TDP‐43 proteinopathy, *C9orf72* expansion, and RNA‐binding protein dysfunction illustrate the molecular overlap between FTD and ALS. Selective vulnerability of different neural networks helps explain their distinct clinical phenotypes [227, 228].

#### 3.3.6 Friedreich's Ataxia

FRDA is a rare autosomal recessive NDD and represents one of the most common inherited ataxias. Unlike aggregation‐centered NDDs discussed above, FRDA is primarily defined by monogenic defects, transcriptional silencing, and mitochondrial metabolic imbalance [110].

Most patients with FRDA carry homozygous GAA trinucleotide repeat expansions in intron 1 of the *FXN* gene. A minority are compound heterozygotes with a GAA expansion on one allele and another pathogenic variant on the other. In the common GAA‐expansion genotype, epigenetic transcriptional repression and impaired transcriptional elongation reduce FXN expression. This loss‐of‐function mechanism distinguishes FRDA from classical proteinopathies driven by toxic protein aggregation [229, 230].

FXN is a mitochondria‐associated protein closely linked to iron–sulfur (Fe–S) cluster biogenesis. FXN deficiency impairs Fe–S protein maturation and the function of associated enzymes. This can reduce ATP production, disrupt redox homeostasis, and sustain energetic stress in tissues with high metabolic demands [231, 232, 233]. Although mitochondrial iron deposition is a hallmark of FRDA, it appears more likely to be a secondary consequence of FXN deficiency and impaired Fe–S biogenesis than the earliest pathogenic driver [232].

Classical pathological studies show that FRDA primarily affects the dorsal root ganglia, large myelinated sensory fibers of the posterior roots, posterior columns, spinocerebellar tracts, and dentate nuclei, rather than beginning as diffuse cortical degeneration. Among these structures, dorsal root ganglion involvement is central because it combines hypoplasia with subsequent progressive degeneration, a distinctive feature of FRDA [234, 235, 236]. Accordingly, although FRDA presents clinically as ataxia, it is more accurately understood as a broader neurodegenerative process. It begins with sensory neuronopathy and subsequently involves long tracts and cerebellar relay structures. This pathological topography indicates that NDDs may also reflect selective vulnerability of long‐range pathways, sensory neurons, and deep nuclei, rather than cortical protein deposition alone.

Beyond the nervous system, FRDA has substantial cardiac and metabolic manifestations, supporting its multisystem nature. Cardiomyopathy, particularly hypertrophic cardiomyopathy, is one of the most prominent extra‐neurological manifestations of FRDA and a major cause of mortality. Abnormalities in glucose metabolism and diabetes are also common [110]. Together, neurological and cardiac involvement reinforce a central feature of FRDA: FXN deficiency preferentially injures tissues with high oxidative and bioenergetic demands.

#### 3.3.7 Multiple Sclerosis

MS is a chronic inflammatory and demyelinating disease of the CNS that also involves neurodegeneration and commonly begins in young adulthood. Compared with protein aggregation‐centered NDDs, MS is primarily characterized by immune‐mediated injury to myelin and oligodendrocytes. Recurrent peripheral inflammatory attacks alone do not explain its long‐term disability. MS is therefore a neuroinflammatory and neurodegenerative disorder in which immune dysregulation, demyelination, remyelination failure, and secondary neuroaxonal degeneration are tightly coupled. Pathologically, MS features multifocal inflammation, demyelination, gliosis, and varying degrees of axonal injury in the white and gray matter of the CNS. Together, these changes form characteristic plaques [110, 237].

MS lesions are dynamic. Some newly formed lesions become inactive or partially repaired, whereas others evolve into chronic active, or smouldering, lesions that sustain low‐grade inflammation and tissue injury at their margins. This lesion heterogeneity is a key pathological feature of MS. Active lesions mainly show peripheral immune‐cell infiltration, BBB disruption, and active demyelination. Chronic active lesions are marked by activated microglia and macrophages at the rim, persistent demyelination in the core and ongoing axonal injury. Pathological and imaging studies further associate chronic active lesions with brain atrophy, irreversible disability accumulation, and disease progression. These lesions are therefore considered an important pathological substrate of progressive tissue injury in MS [131, 238].

Another defining feature of MS is the progressive compartmentalization of inflammation within the CNS. As MS progresses, inflammation becomes less dependent on repeated peripheral immune‐cell entry. It increasingly persists in self‐sustaining inflammatory microenvironments in the meninges, lesion rims, gray matter, and white matter. This process is associated with meningeal inflammation, lymphoid‐like aggregates, cortical demyelination, axonal injury, and cortical neuronal damage. This pattern is particularly relevant to relapse‐independent progression [131, 239, 240].

Gray matter pathology further distinguishes MS among NDDs. MS has traditionally been viewed as a white matter demyelinating disease. However, pathology in cortical and deep gray matter, particularly subpial cortical demyelination, is closely associated with cognitive impairment, progressive disability, and overall disease burden. These gray matter changes are more than passive extensions of white matter lesions. They reflect interactions among meningeal inflammation, local microglial activation, myelin loss, and neuronal vulnerability. Thus, a comprehensive account of MS pathology should include white matter lesions, gray matter injury, and meningeal inflammation [237, 239, 240].

Remyelination failure is another key pathological process underlying sustained MS progression. Some early lesions retain partial repair capacity. However, remyelination is often incomplete or fails because of persistent inflammation, insufficient myelin debris clearance, impaired oligodendrocyte precursor cell differentiation, and a chronically inhibitory microenvironment. As a result, myelin‐mediated protection remains incomplete. Demyelinated axons face increased energy demands, disrupted ion homeostasis, and continued injury [241].

Overall, the classification and pathophysiology of NDDs reflect both shared mechanisms and disease‐specific processes. These mechanisms include pathological protein accumulation, organelle dysfunction, glial reprogramming, and network disconnection. They interact, and their relative contributions vary across disorders. Their combined effects help explain differences in clinical phenotypes, progression rates, and therapeutic responses. Understanding these mechanisms provides the conceptual basis for the following section on clinical features and diagnosis. That section examines how pathological changes give rise to symptom patterns and how biomarkers and neuroimaging can help infer underlying pathology in vivo (Figure 2).

**FIGURE 2:**
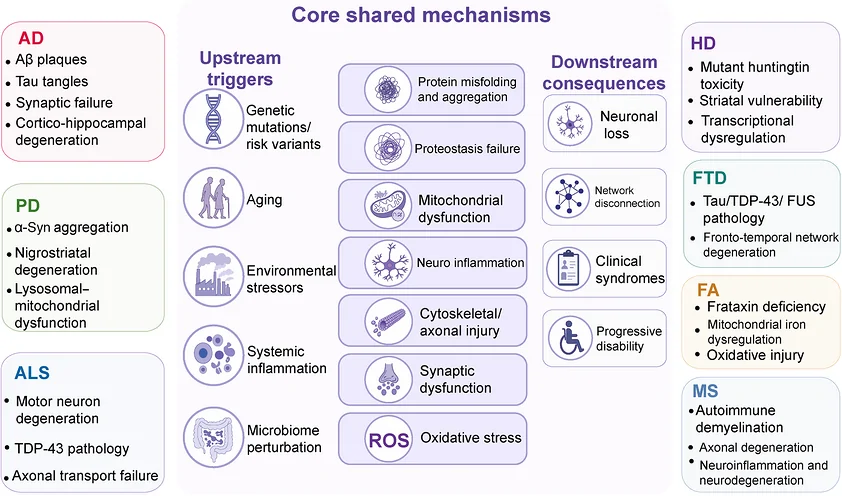
Shared and disease‐specific pathophysiological framework across NDDs. This schematic representation summarizes the convergence and divergence of pathogenic mechanisms across major NDDs. Common upstream triggers, including genetic mutations or risk variants, aging, environmental stressors, systemic inflammation, and microbiome perturbation, converge on core shared mechanisms such as protein misfolding and aggregation, proteostasis failure, mitochondrial dysfunction, neuroinflammation, cytoskeletal and axonal injury, synaptic dysfunction, and oxidative stress. These shared processes contribute to downstream consequences, including neuronal loss, network disconnection, clinical syndromes, and progressive disability. Disease‐specific pathological signatures are shown for AD, PD, ALS, HD, FTD, FA, and MS, highlighting both shared neurodegenerative principles and distinct vulnerable systems. *Abbreviations*: AD, Alzheimer's disease; Aβ, amyloid‐β; α‐syn, α‐synuclein; ALS, amyotrophic lateral sclerosis; FA, Friedreich's ataxia; FTD, frontotemporal dementia; HD, Huntington's disease; MS, multiple sclerosis; NDDs, neurodegenerative diseases; PD, Parkinson's disease; ROS, reactive oxygen species.

## 4 Clinical Features and Diagnosis

The clinical recognition of NDDs is an iterative process that moves from syndromic characterization to etiological inference while incorporating pathobiological evidence. Despite differences in onset, core symptoms, and progression, accurate diagnosis requires systematic integration of clinical history, neuropsychological assessment, neuroimaging, laboratory investigations, and genetic testing as appropriate. This section first outlines general clinical and diagnostic principles across NDDs. It then examines disease‐specific manifestations, diagnostic criteria, and the advantages and limitations of ancillary investigations for AD, PD, ALS, HD, FTD, FRDA, and MS.

### 4.1 General Clinical and Diagnostic Principles

Although NDDs differ in pathological proteins, vulnerable neural systems, and disease trajectories, their clinical recognition follows shared principles. These disorders often begin insidiously and progress gradually, with overlapping cognitive, behavioral, language, motor, sensory, or autonomic manifestations. Therefore, diagnosis is a stepwise process from syndrome recognition to etiological attribution and pathobiological inference, rather than the naming of an isolated symptom.

In clinical practice, the diagnosis of NDDs begins with systematic history‐taking and neurological examination. In patients with cognitive or neurobehavioral changes, assessment should establish the onset, rate of progression, affected domains, and impact on daily functioning. Family and medical histories, psychiatric and behavioral changes, and medication or substance exposures should also be considered to guide syndrome recognition and differential diagnosis [242, 243]. When routine assessment is insufficient, objective cognitive screening and, if necessary, formal neuropsychological evaluation should be used to assess severity and cognitive profiles. These assessments also help distinguish among normal aging, MCI, and established neurodegenerative syndromes [244]. Laboratory testing and structural imaging remain essential for excluding systemic, psychiatric, and metabolic disorders, delirium, and other treatable causes [243].

Ancillary investigations have value at three levels. First, routine laboratory tests exclude alternative or reversible causes, including hypothyroidism, vitamin B12 or folate deficiency, electrolyte disturbances, hepatic or renal dysfunction, syphilis, and human immunodeficiency virus infection [245]. Second, magnetic resonance imaging (MRI), functional imaging, neurophysiology, CSF biomarkers, and blood‐based biomarkers improve etiological specificity and, in some diseases, support early detection [246, 247, 248]. Third, genetic testing is decisive in disorders with clear familial aggregation or monogenic etiology, such as HD and FRDA. In other cases, it mainly supports risk stratification or subtype definition [94, 109].

Clinical diagnosis of NDDs remains challenging because of symptom overlap and heterogeneity. Discrepancies between clinical and gold‐standard neuropathological diagnoses persist even in specialized centers. In a Netherlands Brain Bank study, clinical diagnostic accuracy reached 84% for AD and 83% for FTD. The corresponding Jaccard similarity coefficients were 0.642 and 0.466, respectively, indicating persistent diagnostic ambiguity. The Jaccard coefficients for vascular dementia (VD) and multiple system atrophy (MSA) were 0.117 and 0.465, respectively. These diagnostic discrepancies partly reflect overlapping clinical manifestations across these diseases. Dementia and memory impairment occur in AD, FTD, dementia with Lewy bodies, and VD. Bradykinesia is shared by PD, MSA, progressive supranuclear palsy (PSP), and related disorders. These overlapping features, together with atypical disease trajectories, increase the risk of misdiagnosis when diagnosis relies solely on cross‐sectional phenotypes [249].

### 4.2 Disease‐Specific Features

#### 4.2.1 Alzheimer's Disease

The clinical course of AD is slowly progressive and heterogeneous, involving cognitive impairment, neuropsychiatric symptoms, motor abnormalities, and declining ability to perform activities of daily living. Preclinical AD includes asymptomatic cerebral amyloidosis, Aβ positivity with downstream neuronal injury, and Aβ positivity with neuronal injury plus subtle cognitive decline. This last stage is closest to MCI and carries the highest risk of progression [160]. In the predementia or MCI stage, patients typically show impaired recent memory, difficulty learning new information, reduced ability to plan and organize, and impaired executive function [250]. Neuropsychiatric symptoms such as depression, irritability, nocturnal behavioral changes, social withdrawal, and apathy may emerge before dementia and are associated with progression from normal cognition to MCI and dementia [251, 252]. As AD progresses, cognitive impairment worsens. Beyond memory loss, patients may develop word‐finding difficulty and impaired naming. Increasing naming errors reflect progressive disruption of language processing [253]. Neuroimaging evidence also links naming deficits to early gray matter loss in the left fusiform gyrus, followed by more widespread loss across the left hemisphere [254]. Patients gradually develop disorientation, distractibility, impaired judgment, and reduced illness awareness [255, 256]. Behavioral and psychiatric symptoms also become more prominent, including anxiety, agitation, apathy, sleep disturbance, hallucinations, delusions, aggression, and wandering [257]. In the middle stage, patients may develop ideational and ideomotor apraxia, cortical visual agnosia, and prosopagnosia, alongside a progressive loss of independence and an increasing need for care [258]. In late‐stage AD, higher mental functions are severely impaired. This stage is marked by profound memory loss, marked language decline, apraxia‐related difficulties with eating and swallowing, sphincter dysfunction, and eventual complete dependence on others. Some patients also develop myoclonus, seizures, or extrapyramidal symptoms, most commonly bradykinesia and rigidity. Early extrapyramidal symptoms often indicate faster cognitive and functional decline and a poorer prognosis [259].

The diagnosis of AD has shifted from symptom‐based assessment to an integrated clinical and biological framework. The 1984 National Institute of Neurological and Communicative Disorders and Stroke–Alzheimer's Disease and Related Disorders Association criteria established the classical clinical framework with probable, possible, and definite AD categories. They defined AD as a clinicopathological syndrome, with pathological confirmation required for a definite diagnosis [260]. However, these criteria mainly applied to dementia‐stage disease. They did not cover MCI or preclinical AD or incorporate diagnostically important CSF, positron emission tomography (PET), or MRI biomarkers. Later criteria, including those from the NIA‐AA, retained the clinical category of “probable AD” while extending the concept of AD to a biomarker‐supported disease continuum [158]. The 2024 criteria proposed by Jack et al. further revised the diagnostic framework for AD. In this framework, an abnormal Core 1 biomarker result is sufficient to establish an AD diagnosis. Core 1 measures include amyloid PET, CSF Aβ42/40, CSF phosphorylated tau at threonine 181 (p‐tau181)/Aβ42, CSF t‐tau/Aβ42, and plasma assays with performance comparable to approved CSF tests, particularly selected plasma phosphorylated tau at threonine 217 (p‐tau217) assays. These markers correspond to the earliest detectable in vivo stage of AD and allow asymptomatic individuals to be classified within the AD disease continuum. By contrast, Core 2 biomarkers mainly reflect later tau pathological burden. They are used for biological staging and progression‐risk assessment, and to increase confidence that symptoms are attributable to AD. They do not serve as standalone diagnostic evidence. Importantly, biological diagnosis does not replace clinical judgment. Comorbid pathologies and factors such as near‐threshold variations, head trauma, renal dysfunction, and other neurological diseases may affect interpretation. In particular, an A−T2+ profile does not support AD and is more suggestive of non‐AD tau pathology [246].

#### 4.2.2 Parkinson's Disease

The clinical presentation of PD is heterogeneous, encompassing motor and nonmotor symptoms. According to the MDS clinical diagnostic criteria, the core motor syndrome is bradykinesia accompanied by rest tremor and/or rigidity. Motor symptoms usually begin asymmetrically in one limb and later progress bilaterally [261]. Patients may also develop masked facies, micrographia, slowed fine motor movements, difficulty turning, and a shuffling gait. With progression, postural instability, freezing of gait, and balance impairment become major causes of falls, restricted mobility, and loss of independence [2, 262]. The clinical spectrum of PD is not limited to motor dysfunction. Nonmotor symptoms are common and may precede motor symptoms by several years. They continue to evolve throughout the disease course. Common nonmotor manifestations include hyposmia, constipation, RBD, depression, anxiety, fatigue, pain, and autonomic dysfunction. Autonomic dysfunction includes orthostatic hypotension, urinary dysfunction, and sexual dysfunction. With further progression, cognitive impairment, hallucinations, and psychiatric symptoms become more prominent in some patients. These manifestations increase caregiving burden and often impair quality of life to a greater extent than motor symptoms alone [263, 264].

The diagnosis of PD remains primarily clinical. The 2015 MDS criteria first require motor parkinsonism, defined by bradykinesia with rest tremor or rigidity. Absolute exclusion criteria, red flags, and supportive criteria are then used to classify the diagnosis as clinically established or clinically probable PD [261]. A multicenter validation study found that the MDS criteria for clinically probable PD achieved 92.6% overall accuracy, 94.5% sensitivity, and 88.5% specificity against expert clinical diagnosis. These criteria outperformed the UK Brain Bank criteria [265]. In practice, diagnosis should integrate history‐taking, neurological examination, and longitudinal follow‐up. Levodopa responsiveness increases diagnostic confidence, but diagnostic accuracy generally improves with disease duration, especially after more than 5 years of follow‐up. Diagnosis should therefore not rely solely on baseline drug response [266]. Ancillary investigations are more useful for differential diagnosis than for confirming PD. Routine brain MRI is often nonspecific in typical PD. It can help exclude secondary causes, such as vascular parkinsonism and normal‐pressure hydrocephalus, and identify imaging clues to atypical parkinsonian syndromes, such as MSA and PSP [267]. Dopamine transporter single‐photon emission computed tomography can demonstrate a nigrostriatal dopaminergic deficit in patients with diagnostically ambiguous tremor or parkinsonism. This can help distinguish PD from essential tremor and functional parkinsonism [267, 268]. However, DAT‐SPECT has limited ability to distinguish PD from atypical parkinsonism because these syndromes may also show presynaptic dopaminergic dysfunction [269]. The α‐syn seed amplification assay (αSyn‐SAA) is among the most promising diagnostic methods. Recent reviews and meta‐analyses report an overall sensitivity of approximately 86% and specificity of approximately 92% for αSyn‐SAA in distinguishing PD from controls [270]. However, assay platforms, sample types, interpretation criteria, and data‐sharing frameworks still require harmonization. αSyn‐SAA has not replaced the routine clinical diagnostic pathway [271]. Beyond αSyn‐SAA, exploratory blood‐based sensing platforms have also been developed for PD‐related biomarker detection. These include electrochemical glycoprotein sensors and assays for glycoprotein nonmetastatic melanoma protein B. These approaches remain at the method‐development stage and require independent clinical validation [272].

#### 4.2.3 Amyotrophic Lateral Sclerosis

ALS is characterized by progressive upper and lower motor neuron involvement, usually with focal onset followed by spread to adjacent or distant regions. Initial manifestations commonly include unilateral limb weakness, impaired fine hand movements, foot drop, fasciculations, muscle atrophy, spasticity, hyperreflexia, and pathological reflexes [196, 273]. The combination of upper and lower motor neuron signs helps distinguish ALS from many peripheral nerve or muscle diseases. ALS is commonly classified by site of onset as limb‐onset, bulbar‐onset, or respiratory‐onset disease. Limb‐onset ALS is the most common form. Bulbar‐onset ALS presents with dysarthria, dysphagia, hypernasal speech, laryngospasm, or sialorrhea and is associated with faster decline and a poorer prognosis [196]. Respiratory‐onset ALS is uncommon but clinically important. Early dyspnea, nocturnal hypoventilation, morning headache, or orthopnea may be mistaken for primary respiratory or sleep disorders [274, 275, 276]. As ALS progresses, motor impairment extends to speech, swallowing, respiration, and axial function. Bulbar dysfunction increases the risk of aspiration and nutritional compromise, while respiratory muscle involvement becomes a major direct cause of death.

ALS also includes nonmotor and cognitive‐behavioral manifestations that contribute substantially to disease burden. Common nonmotor symptoms include pain, fatigue, sleep disturbance, mood changes, and pseudobulbar affect (PBA) [277, 278]. ALS and FTD are now understood as part of a spectrum with shared clinical, genetic, and pathological features. Approximately 50% of patients with motor neuron disease develop cognitive or behavioral abnormalities, and about 10–15% meet diagnostic criteria for FTD [279]. These changes mainly affect executive function, language, social cognition, and behavioral regulation. This pattern supports systematic neuropsychological and behavioral screening during ALS assessment.

ALS diagnosis remains primarily clinical and depends on identifying progressive upper and lower motor neuron involvement while excluding mimics. Because no single definitive test exists, diagnostic evaluation integrates history‐taking, repeated neurological examinations, electromyography (EMG), and ancillary investigations. Current approaches emphasize early recognition of progressive motor neuron disease, without waiting for a fully developed phenotype. Diagnostic delays remain common and limit timely supportive care, trial enrolment, and early intervention [280, 281].

The Gold Coast criteria are an important framework for ALS recognition, emphasizing clinical feasibility and sensitivity at earlier disease stages. Their core requirements are progressive motor impairment after previously normal motor function, clinical or electrophysiological evidence of upper and lower motor neuron dysfunction, and exclusion of alternative disorders [280, 282]. In practice, these criteria provide an entry point for diagnosis and should be applied alongside a complete neuromuscular evaluation. Needle EMG remains central to diagnosis because it can detect diffuse lower motor neuron involvement that is not clinically apparent. This helps confirm the multiregional spread characteristic of ALS [247]. Brain and spinal MRI, laboratory tests, and selected neuroimaging or genetic assessments are mainly used to exclude mimics, including structural, neuropathic, neuromuscular, metabolic, inflammatory, and immune‐mediated disorders. When cognitive or behavioral abnormalities, PBA, or frontotemporal features are present, evaluation should extend beyond the motor system and include neuropsychological and behavioral screening within the ALS‐FTD continuum [86]. Beyond clinical criteria and electrophysiology, fluid biomarkers are increasingly important in ALS diagnosis. Among these, neurofilaments, particularly neurofilament light chain (NfL), are well‐supported biomarkers. NfL is elevated in ALS, has diagnostic and prognostic value, and is approaching clinical translation [283, 284]. However, current biomarkers still complement rather than replace clinical and electrophysiological diagnosis.

#### 4.2.4 Huntington's Disease

HD is best understood as a progressive continuum involving motor, cognitive, psychiatric, and behavioral abnormalities, rather than a linear sequence that always begins with chorea. Although most patients develop symptoms in adulthood, subtle oculomotor abnormalities, impaired coordination, executive dysfunction, and mood or behavioral changes may appear during the prodromal phase. Chorea is the most characteristic motor feature. As the disease progresses, bradykinesia, dystonia, rigidity, gait instability, dysarthria, and dysphagia contribute increasingly to motor impairment and functional decline. Cognitive impairment, especially executive dysfunction involving attention, processing speed, organization, planning, and cognitive flexibility, may precede overt motor symptoms and is associated with disease progression and functional decline [94, 285]. Psychiatric and behavioral symptoms may also precede motor abnormalities and include depression, anxiety, irritability, apathy, obsessive‐compulsive symptoms, and aggression. Apathy is particularly common and often worsens with progression. Psychotic symptoms increase the complexity of care and functional burden [94, 286].

The diagnosis of HD is based on clinical assessment and molecular genetic confirmation. In patients with progressive motor abnormalities, cognitive decline, or psychiatric or behavioral changes, detection of a pathogenic CAG repeat expansion in *HTT* establishes the diagnosis. Family history and neuroimaging can strengthen clinical suspicion but cannot provide confirmation [94, 287]. For asymptomatic adults at familial risk, predictive genetic testing should be performed with genetic counseling and psychological assessment rather than as routine screening [288]. The Unified Huntington's Disease Rating Scale (UHDRS) is widely used to assess motor, cognitive, behavioral, and functional status. It supports severity assessment and longitudinal follow‐up but does not replace genetic confirmation [289]. With advances in prodromal research, HD diagnosis and staging are moving beyond late clinical phenotypes to encompass a continuum integrating genetic status, pathobiology, early clinical signs, and functional decline. The HD‐ISS embodies this transition and is particularly useful for defining participant groups in prodromal studies and disease‐modifying treatment trials [208]. Ancillary tools can support HD assessment. Structural MRI may reveal caudate and putaminal atrophy before a definite motor diagnosis. Magnetic resonance spectroscopy and fluid biomarkers may also detect early or prodromal abnormalities [290, 291, 292]. However, these measures remain sources of supportive evidence, stratification tools, or research endpoints rather than independent criteria for routine diagnosis. Differential diagnosis is essential when family history is absent, age at onset is atypical, or clinical features are nonclassical. HD‐like syndromes and acquired choreas may mimic HD [293, 294].

#### 4.2.5 Frontotemporal Dementia

FTD is a group of clinical syndromes characterized by selective degeneration of the frontal and anterior temporal lobes. Compared with AD, FTD is more closely associated with young‐onset dementia and is clinically heterogeneous, with bvFTD and PPA‐related syndromes as the two core phenotypic categories [5, 295].

bvFTD is the most representative subtype and often presents early with behavioral disinhibition, apathy, loss of empathy, stereotyped or compulsive behaviors, dietary changes, and executive dysfunction rather than prominent memory loss. Under the revised diagnostic criteria, possible bvFTD requires progressive behavioral and/or cognitive decline and at least three of six core features. Probable bvFTD additionally requires functional decline and supportive frontal and/or anterior temporal imaging abnormalities [296].

Language‐predominant FTD is mainly recognized through the PPA framework. PPA is divided into nonfluent/agrammatic, semantic, and logopenic variants, with the first two more typical of the FTD spectrum. The nonfluent/agrammatic variant features effortful, halting, and grammatically impaired speech, often with apraxia of speech. The semantic variant is characterized by impaired naming and single‐word comprehension, reflecting a progressive loss of semantic knowledge [297].

Early FTD symptoms may overlap with those of primary psychiatric disorders, atypical AD, or other frontal network diseases. This is especially challenging in bvFTD, where apathy, impulsivity, emotional blunting, and impaired social judgment may be misinterpreted as depression, bipolar disorder, or personality changes. Diagnosing FTD therefore requires an integrated assessment of informant history, disease progression, neuropsychological testing, and neuroimaging findings. A single outpatient impression is insufficient [298, 299].

MRI remains the primary imaging tool at baseline for FTD. Fluorodeoxyglucose PET (FDG‐PET) may increase diagnostic confidence when structural imaging is inconclusive. Unlike AD, FTD lacks highly specific fluid biomarkers that directly define the disease. Diagnosis therefore remains primarily syndrome‐based, with biomarkers serving as adjunctive stratification tools [298]. Serum NfL may help distinguish bvFTD from primary psychiatric disorders, although its performance is age‐dependent [300]. Genetic testing is important when disease onset is early, family history is positive, or genetic FTD is suspected. *C9orf72*, *MAPT*, and *GRN* are the principal causative genes [295].

#### 4.2.6 Friedreich's Ataxia

FRDA is an autosomal recessive multisystem disorder characterized mainly by progressive neurological involvement, with additional cardiac, skeletal, and metabolic manifestations. Typical onset occurs in late childhood or adolescence, but the clinical spectrum extends beyond the classical childhood‐onset ataxia phenotype. Progressive gait instability is usually the earliest manifestation, followed by upper‐limb ataxia, dysarthria, dysphagia, peripheral sensory and motor nerve involvement, pyramidal signs, and spasticity. The combination of progressive ataxia, reduced or absent lower‐limb tendon reflexes, impaired vibration and position sense, and extensor plantar responses provides key diagnostic clues [109, 110].

Clinical recognition of FRDA must account for systemic manifestations, including scoliosis, pes cavus, hypertrophic cardiomyopathy, diabetes, and impaired glucose tolerance. FRDA may also present atypically, with later onset, slower progression, partially preserved reflexes, or milder systemic complications. Such presentations can mimic other inherited or spastic ataxia syndromes. Clinical suspicion therefore depends on recognizing a characteristic constellation of progressive gait instability, impaired deep sensation, reduced lower‐limb reflexes, and pyramidal signs [109]. Initial presentation to orthopedic or cardiology services may further delay recognition.

In modern diagnostic frameworks, FRDA is a molecularly confirmed genetic disease. In individuals with suggestive clinical features, diagnosis is established by identifying biallelic pathogenic variants in the *FXN* gene. Most patients carry biallelic GAA repeat expansions in intron 1. A minority have a GAA expansion on one allele and a sequence variant or large deletion on the other [301]. Because this pathogenic repeat is intronic, conventional exome sequencing or standard next‐generation sequencing may miss it. Clinical testing must therefore include assays targeting the *FXN* GAA repeat expansion. If only one expanded allele is detected, sequence‐level and copy‐number analysis should follow.

Ancillary investigations mainly support phenotypic characterization, differential diagnosis, and multisystem assessment rather than replacing genetic confirmation. Brain MRI may show normal cerebellar volume or only mild changes, whereas spinal cord atrophy is more suggestive of FRDA. Normal imaging therefore does not exclude FRDA [302, 303]. Neurophysiological testing commonly shows sensory‐predominant neuropathy with reduced or absent sensory nerve action potentials and relatively preserved motor conduction [302, 304]. Cardiac assessment is essential. An electrocardiogram and echocardiography should be performed at diagnosis and during follow‐up to assess cardiomyopathy or arrhythmia‐related manifestations [305, 306]. Postdiagnostic follow‐up should also include neurological, cardiac, endocrine, orthopedic, and ophthalmological assessments and, when indicated, audiological assessment.

#### 4.2.7 Multiple Sclerosis

MS is a chronic immune‐mediated inflammatory demyelinating disease of the CNS, accompanied by varying degrees of axonal and neuronal injury. Its clinical course is heterogeneous, ranging from a relapsing‐remitting pattern to gradual functional decline beginning at disease onset. Typical initial manifestations include optic neuritis, partial myelitis, and brainstem or cerebellar syndromes. Depending on lesion location, patients may also have sensory disturbances, limb weakness, ataxia, diplopia, or bladder dysfunction. Thus, clinical recognition is guided by the pattern of CNS involvement across anatomical sites and over time, rather than by any single symptom [307, 308].

Unlike disorders that rely more heavily on molecular pathology or genetic confirmation, MS diagnosis requires integrated clinical, imaging, and laboratory assessment. Diagnosis relies on characteristic CNS findings, including dissemination in space (DIS) and, when required, time, after excluding more plausible alternatives. White matter lesions or positive CSF findings alone do not establish MS [248, 307, 309].

MRI remains central in the current diagnostic framework. The 2024 revisions to the McDonald criteria include the optic nerve as a fifth DIS site. They also allow the central vein sign, paramagnetic rim lesions, and CSF κ free light chains to support diagnostic specificity in selected contexts [248, 310]. These changes improve early recognition but require careful interpretation to avoid overdiagnosis in older patients, those with vascular risk factors, or those with atypical presentations.

CSF testing remains an important adjunct, with oligoclonal bands (OCBs) serving as an established marker of intrathecal IgG synthesis [311]. Under the 2024 revised criteria, CSF κ free light chains have also been incorporated as supportive evidence to increase diagnostic confidence in selected situations [248]. However, positive CSF findings are not specific to MS and must be interpreted in the context of the clinical presentation and the morphology and distribution of MRI lesions.

A central diagnostic challenge is the broad differential diagnosis of MS. Misdiagnosis often results from overinterpreting MRI white matter lesions, CSF findings, or a history suggestive of demyelination when the clinical presentation is atypical [309]. Priority differential diagnoses include AQP4‐IgG‐positive neuromyelitis optica spectrum disorder (AQP4‐NMOSD), myelin oligodendrocyte glycoprotein antibody‐associated disease, vascular or migraine‐related white matter abnormalities, hereditary small‐vessel disease, granulomatous or neoplastic disorders, and other inflammatory or infectious CNS diseases [309, 312, 313].

In summary, the diagnosis of NDDs is shifting from symptom‐triggered recognition toward the integration of biological evidence. AD diagnosis increasingly incorporates fluid and imaging biomarkers. αSyn‐SAA and NfL provide diagnostic support in PD and ALS, respectively. HD and FRDA remain dependent on genetic confirmation. FTD and MS still rely primarily on clinical syndromes supported by imaging and other ancillary evidence. Accurate diagnosis is essential for prognostication and informs therapeutic decisions, including the strategies discussed in the next chapter (Figure 3).

**FIGURE 3:**
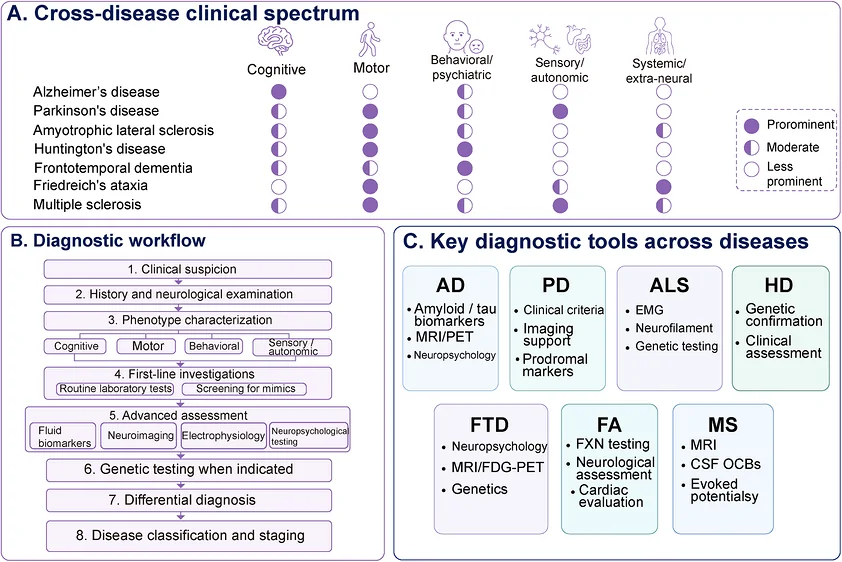
Clinical manifestations and diagnostic framework across major NDDs. (A) Major NDDs exhibit overlapping but distinct clinical spectra across cognitive, motor, behavioral/psychiatric, sensory/autonomic, and systemic domains. (B) Despite phenotypic heterogeneity, diagnostic evaluation generally follows a common workflow beginning with clinical suspicion and neurological assessment, followed by phenotype characterization and multimodal investigations, including fluid biomarkers, neuroimaging, electrophysiology, neuropsychological testing, and genetic testing when appropriate. (C) The relative importance of specific diagnostic tools differs across diseases, with biomarker‐supported diagnosis in AD, imaging and phenotypic support in PD, electrophysiology and neurofilament assessment in ALS, genetic confirmation in HD and FA, neuropsychological and imaging‐based evaluation in FTD, and MRI‐ and CSF‐based assessment in MS. *Abbreviations*: AD, Alzheimer's disease; ALS, amyotrophic lateral sclerosis; CSF, cerebrospinal fluid; EMG, electromyography; FA, Friedreich's ataxia; FDG‐PET, fluorodeoxyglucose positron emission tomography; FTD, frontotemporal dementia; HD, Huntington's disease; MRI, magnetic resonance imaging; MS, multiple sclerosis; NDDs, neurodegenerative diseases; OCBs, oligoclonal bands; PD, Parkinson's disease; PET, positron emission tomography.

## 5 Pharmacological and Emerging Therapeutic Strategies

The therapeutic landscape for NDDs is shifting from symptomatic control toward disease‐modifying interventions targeting core pathological mechanisms. These include gene therapy, cell therapy, nucleic acid‐based therapeutics, immunomodulation, and multitarget small molecules. However, the maturity of clinical evidence varies across disorders. Antiamyloid antibodies for AD, *SOD1*‐targeted antisense therapy for ALS, a nuclear factor erythroid 2‐related factor 2 (Nrf2) activator for FRDA, and high‐efficacy immunomodulatory therapies for MS have entered clinical practice. PD, HD, and FTD still rely largely on symptomatic management, while disease‐modifying options remain limited for some forms of progressive MS. This chapter summarizes established and emerging strategies for each disorder. Section 5.8 then compares representative clinical evidence.

### 5.1 Alzheimer's Disease

#### 5.1.1 Established Therapeutic Strategies

Current AD pharmacotherapy includes two complementary approaches: symptomatic drugs and antiamyloid disease‐modifying therapy for patients with biomarker‐confirmed early‐stage AD. No established treatment is curative, and clinical decision‐making depends on disease stage, biological eligibility, monitoring capacity, comorbidities, and available care support.

Symptomatic pharmacotherapy remains foundational in routine AD management. Cholinesterase inhibitors, including donepezil, galantamine and rivastigmine, are recommended as first‐line monotherapy for mild‐to‐moderate AD [314, 315, 316]. Memantine is mainly used for moderate AD when cholinesterase inhibitors are not tolerated or contraindicated, and for severe AD [314]. These drugs mainly help maintain cognitive and functional stability rather than block neurodegeneration.

Antiamyloid monoclonal antibodies mark an important shift in AD therapy. Lecanemab [317] and donanemab [318, 319] are indicated for AD and should be initiated in patients with MCI or mild dementia after confirmation of Aβ pathology. AD treatment is therefore shifting from symptom‐based management toward biomarker‐guided early identification and intervention.

Established AD treatment also includes individualized supportive care to preserve function, relieve symptoms and address long‐term care needs. For behavioral and psychological symptoms, clinicians should identify underlying triggers and prioritize psychosocial and environmental interventions. Antipsychotics should be used only when necessary, for a short period and with caution [320]. These supportive measures cannot replace disease‐modifying therapy. The limited benefits of current symptomatic drugs and approved anti‐Aβ therapies justify continued development of emerging treatments.

#### 5.1.2 Emerging Therapeutic Strategies

Beyond established treatments, emerging AD therapies include next‐generation Aβ‐targeted strategies, tau‐directed therapies, multitarget small molecules, and cell‐based approaches. However, the strength of evidence varies, and most candidates should still be regarded as exploratory until confirmatory efficacy and long‐term safety data become available.

Aβ‐centered research is extending beyond approved antibodies toward next‐generation antibodies and active immunotherapies. Remternetug is being evaluated in Phase III studies of early and preclinical AD, but remains investigational until confirmatory results are available [321]. Among active immunotherapies, ABvac40 had an acceptable safety and tolerability profile and induced sustained antibody responses in a Phase II study, although its clinical efficacy has not been established [322]. Vaccine‐based strategies may offer advantages in durability and scalability, but their clinical value requires further validation.

Tau is an attractive target because the burden of tau pathology correlates closely with neurodegeneration and clinical deterioration. However, tau‐directed therapies have not consistently produced clinical benefits. In the Phase IIa TOGETHER study, bepranemab did not meet its primary Clinical Dementia Rating–Sum of Boxes (CDR‐SB) endpoint. Secondary analyses suggested possible benefit in selected subgroups, particularly patients with lower baseline tau burden or *APOE* ε4 noncarriers [323]. ACI‐35.030 was well tolerated in Phase Ib/IIa testing and induced sustained antibody responses against phosphorylated tau species, supporting further Phase IIb evaluation [324].

Multitarget small molecules remain attractive because AD progression involves proteostasis failure, neuroinflammation, synaptic dysfunction, and toxic protein accumulation. Buntanetap is thought to reduce the translation of APP, tau, and α‐syn, proteins implicated in neurodegenerative pathology. In a Phase II/III study, buntanetap was generally safe and well tolerated. The prespecified efficacy endpoints were not met, but a posthoc analysis found a nominally significant improvement from baseline in ADAS–Cog11 scores among biomarker‐positive patients with mild AD [325]. A Phase III trial in early AD is ongoing [326]. Nevertheless, buntanetap remains investigational because the reported cognitive benefit comes from a posthoc subgroup analysis and has yet to be confirmed.

Cell therapy is a distinct emerging strategy that aims to modulate the AD brain microenvironment by reducing inflammation, providing trophic support, promoting repair and limiting atrophy [327]. Mesenchymal stem cell (MSC)‐based approaches have attracted attention, with laromestrocel as a representative example [328]. In a Phase IIa trial in mild AD, laromestrocel met its primary safety endpoint. The study also reported favorable secondary signals for composite AD scores, brain volume loss, hippocampal atrophy, and neuroinflammation‐related imaging measures. However, its small sample size leaves efficacy unproven [329].

Despite progress, AD therapies remain constrained by modest clinical benefits, safety concerns, and practical barriers to use. Anti‐Aβ monoclonal antibodies slow cognitive and functional decline, but provide only a modest delay in disease progression and do not reverse established neuronal injury. Follow‐up in pivotal trials also remains short relative to the long natural history of AD [330, 331]. Their real‐world use is also limited by amyloid‐related imaging abnormalities (ARIA), repeated infusions, serial MRI monitoring and strict patient selection. In clinical trials of lecanemab and donanemab, *APOE* ε4 homozygosity was the strongest risk factor for ARIA, particularly severe radiographic ARIA with hemosiderin deposition (ARIA‐H) [317, 332]. Therefore, trial efficacy should not be directly equated with effectiveness in routine care, especially in older patients with comorbidities or imaging evidence suggestive of cerebral amyloid angiopathy.

Emerging therapies face similar limitations. For tau‐targeted therapies, the key challenge is translating immunogenicity, target engagement, or biomarker changes into reproducible clinical benefits. Current results remain inconsistent, with possible benefits often limited to selected subgroups or settings [333]. Multitarget small molecules and cell therapies also remain at an exploratory stage because available evidence is largely derived from early‐stage studies, subgroup signals, or secondary outcomes and does not establish definitive efficacy. For cell therapy, additional barriers include dose standardization, durability, manufacturing consistency, delivery route, immunological risk, and cost. Overall, short‐term biomarker or imaging improvements should not be assumed to represent durable and functionally meaningful clinical benefits.

### 5.2 Parkinson's Disease

#### 5.2.1 Established Therapeutic Strategies

Established PD therapies remain centered on symptomatic control, aiming to improve motor symptoms, reduce complications, alleviate nonmotor burden, and preserve functional independence. Although disease‐modifying strategies targeting α‐syn, inflammation, mitochondrial dysfunction, and genetic mechanisms are advancing, no intervention is yet widely accepted as slowing PD progression. Established management therefore relies on dopaminergic replacement, adjunctive pharmacotherapy, device‐aided treatment, rehabilitation, and supportive care [262, 334].

Levodopa remains the mainstay of pharmacological treatment and the most effective drug for PD motor symptoms. The American Academy of Neurology guideline recommends levodopa over dopamine agonists or monoamine oxidase B (MAO‐B) inhibitors for patients with early‐stage PD who require dopaminergic therapy. It also recommends immediate‐release levodopa as the initial formulation [335]. European guidelines further emphasize individualizing initial monotherapy according to age, biological reserve, comorbidities, psychiatric or behavioral risk, and patient preference. MAO‐B inhibitors or nonergot dopamine agonists may be considered for younger patients concerned about long‐term motor complications. Levodopa remains preferred when faster or stronger symptom relief is needed [266].

With longer disease duration and chronic levodopa use, many patients develop wearing‐off and other OFF episodes, as well as dyskinesia. Treatment then shifts toward pharmacokinetic optimization and reduction of pulsatile dopaminergic stimulation. Evidence‐based reviews support several adjunctive options for motor fluctuations, including dopamine agonists, catechol‐O‐methyltransferase (COMT) inhibitors, MAO‐B inhibitors, amantadine formulations, infusion therapies, and selected deep brain stimulation (DBS) approaches [336]. COMT inhibitors and MAO‐B inhibitors mainly prolong levodopa benefit and reduce OFF time, whereas amantadine has an established role in levodopa‐induced dyskinesia [266].

For patients with persistent motor fluctuations, dyskinesia, or refractory tremor despite optimized pharmacotherapy, device‐aided therapies should be considered. DBS is the most established surgical strategy. Subthalamic nucleus DBS (STN‐DBS) has strong evidence for improving motor symptoms, reducing OFF time and enhancing quality of life. Globus pallidus internus DBS (GPi‐DBS) remains an important alternative [336]. In addition, rehabilitative interventions, including exercise training, physical therapy, and speech and swallowing therapy, remain important components of long‐term management. Their primary role is to maintain function rather than modify disease progression [266, 337].

#### 5.2.2 Emerging Therapeutic Strategies

Emerging PD therapies are moving beyond traditional dopamine replacement toward optimized symptomatic control, multitarget molecular modulation, gene therapy, and cell replacement‐based repair.

Emerging symptomatic strategies focus on more continuous drug delivery and individualized neuromodulation. Continuous subcutaneous foscarbidopa/foslevodopa infusion has been shown to improve motor fluctuations and is approved for this indication in advanced PD [338, 339]. It provides more stable levodopa exposure without relying on further peak‐dose escalation. Adaptive DBS (aDBS) may improve motor symptoms and quality of life compared with conventional continuous stimulation. However, the evidence comes from small, device‐dependent studies and requires validation in larger trials [340, 341].

Multitarget small molecules aim to modulate several PD‐related processes, including protein aggregation, inflammation, synaptic dysfunction, and organelle homeostasis. Buntanetap is thought to reduce the translation of multiple neurotoxic proteins through iron‐responsive elements in their messenger RNAs (mRNAs). Early AD and PD studies have reported a favorable safety profile and preliminary clinical signals [342]. Glucagon‐like peptide‐1 (GLP‐1) receptor agonists and strategies targeting *GBA1*‐related lysosomal dysfunction also remain active areas of research. In a Phase II randomized trial involving patients with early PD, lixisenatide was associated with less progression of motor disability over 12 months than placebo. Gastrointestinal adverse effects were frequent, and further trials with larger samples and longer follow‐up are needed to clarify its efficacy and safety [343]. As a strategy for *GBA1*‐related lysosomal dysfunction, ambroxol showed an acceptable safety profile and target engagement in a PD dementia trial, but did not improve cognition [344].

Gene‐based and nucleic acid‐based therapies are expanding beyond neurotrophic factor delivery to include molecularly stratified interventions. After bilateral intraputaminal delivery, an adeno‐associated virus (AAV) serotype 2 vector encoding glial cell line‐derived neurotrophic factor (GDNF), designated AAV2‐GDNF, was well tolerated, with clinical measures showing numerical stability in mild PD and numerical improvement in moderate PD [345]. The first‐in‐human Phase I trial of the *LRRK2*‐targeted antisense oligonucleotide (ASO) BIIB094/ION859 demonstrated target engagement in patients with PD, moving molecular‐lowering strategies into clinical testing [346].

Cell therapy has substantial reparative potential in PD treatment. Early clinical studies using human embryonic stem cell‐derived or induced pluripotent stem cell (iPSC)‐derived dopaminergic progenitors suggest graft survival and dopamine production, with no tumors detected during short‐term follow‐up. These findings provide preliminary human safety evidence for cell replacement therapy [347, 348]. A Phase I/IIa study of purified human embryonic stem cell‐derived A9 dopaminergic progenitors also showed acceptable safety and exploratory clinical signals [349].

Biologics targeting pathological proteins remain an important research area, with α‐syn monoclonal antibodies as the main example. Cinpanemab did not slow disease progression in early PD. Prasinezumab trials have also failed to meet their primary endpoints [350, 351]. Thus, although α‐syn remains a biologically plausible target, translating this rationale into clinical benefit remains challenging.

Despite mechanistic expansion and early clinical signals, emerging PD therapies still face major evidentiary limitations. Most therapies remain in early‐ or mid‐stage development. Their studies often have small samples, short follow‐up, and endpoints centered on exploratory biomarkers, motor scores, or imaging signals. Differences in population stratification, concomitant therapies, endpoints, and statistical assumptions further limit comparisons across trials and generalization to clinical practice [334].

Safety and risk‐benefit issues also limit translation. For small molecules and biologics, adverse‐event burden must be weighed against the magnitude of benefit. For gene and cell therapies, unresolved issues include CNS delivery, dose control, long‐term expression or sustained suppression, immune responses, immunosuppression, durability, and the need for prolonged follow‐up.

Practical barriers also remain substantial. Many emerging therapies depend on specific genetic backgrounds, biomarkers, or disease stages, limiting eligible populations. Gene therapy, cell therapy, and aDBS require complex screening, procedures, and long‐term monitoring. aDBS additionally requires device management. These demands create implementation barriers, increase costs, and raise equity concerns. For progressive NDDs, irreversible interventions, immunosuppression, and delayed adverse events must be carefully weighed against short‐term biological or exploratory clinical signals.

### 5.3 Amyotrophic Lateral Sclerosis

#### 5.3.1 Established Therapeutic Strategies

Established ALS treatment aims to slow disease progression, preserve function, reduce symptom burden, and improve quality of life, but routine clinical options remain limited. The 2024 European Academy of Neurology (EAN) guideline recommends lifelong riluzole from diagnosis and first‐line tofersen for progressive ALS caused by pathogenic *SOD1* variants. It does not recommend edaravone outside clinical trials [352].

Riluzole remains the foundational standard drug for ALS. Randomized trials have shown that riluzole prolongs survival and modestly slows aspects of progression [353]. The 2024 EAN guideline recommends lifelong use from diagnosis [352]. Liver function and tolerability should be monitored during treatment. Although its benefit is modest, riluzole remains central because of its consistent evidence base, accessibility, and long‐term clinical experience.

Edaravone is approved for ALS in some regions and may be considered for selected patients. However, the 2024 EAN guideline does not recommend its use outside clinical trials [352]. In a pivotal trial involving carefully selected patients with early disease and preserved function, edaravone slowed decline in ALSFRS‐R scores [354]. Compared with riluzole, edaravone's evidence comes from a more narrowly defined trial population, and its clinical use varies by region.

For ALS caused by pathogenic *SOD1* variants, tofersen represents the first approved genotype‐guided therapy for ALS. It received accelerated approval for *SOD1*‐associated ALS based on a reduction in plasma NfL [355]. In long‐term follow‐up, earlier treatment was associated with numerically less decline in respiratory function, muscle strength, and quality of life than delayed treatment [356]. Although it applies to a small genetic subgroup and further outcome evidence is needed, tofersen represents an important precision‐medicine approach to ALS treatment.

Supportive care and multidisciplinary management remain indispensable in established ALS treatment. The 2024 EAN guideline emphasizes nutritional support, swallowing management, noninvasive ventilation, airway secretion control, and communication assistance. It also addresses symptom control and psychological and palliative care [352]. Because individual drugs offer limited benefits, ALS treatment should combine disease‐modifying pharmacotherapy with long‐term supportive care.

Treatments already used in clinical practice should be reassessed when new trial evidence emerges. AMX0035 was initially approved for ALS. The subsequent Phase III PHOENIX trial failed to meet its primary endpoint [357], and the company later withdrew the drug from the US and Canadian markets [358]. AMX0035 therefore illustrates why an initially approved therapy must be reassessed after a negative confirmatory trial.

#### 5.3.2 Emerging Therapeutic Strategies

Emerging ALS therapies increasingly focus on mechanism‐driven and molecularly stratified strategies, including gene therapy, anti‐inflammatory approaches, modulation of proteostasis and cellular stress, targeted neuroprotection, and cell‐based therapy.

Gene therapy aims to reduce pathogenic transcripts or correct molecular abnormalities in genetically defined ALS subtypes. Following the development of tofersen for SOD1‐associated ALS, ulefnersen (jacifusen) has emerged as a closely watched ASO strategy for FUS‐associated ALS. A 2025 case series reported reduced CSF NfL after jacifusen treatment and reduced FUS protein in postmortem tissue from four participants. Possible slowing of disease progression was observed in two participants, providing preliminary evidence for individualized treatment in rare genetic ALS [359].

Neuroinflammation contributes to ALS progression, making anti‐inflammatory approaches an important area of research. Masitinib, a tyrosine kinase inhibitor with anti‐inflammatory and neuroprotective potential, has shown a possible slowing of functional decline when combined with riluzole in selected patients [360]. Complement inhibition is another emerging approach, although its clinical value in ALS remains uncertain.

Dysregulated proteostasis and cellular stress, including TDP‐43‐associated stress granules and activation of the integrated stress response, are key mechanisms in ALS. DNL343 activates eukaryotic translation initiation factor 2B (eIF2B) and modulates the integrated stress response. Its ability to promote stress granule disassembly has been demonstrated in preclinical models, while clinical benefit in ALS remains unproven [361]. TPN‐101 is another molecularly targeted candidate for *C9orf72*‐associated ALS/FTD. It is designed to inhibit long interspersed nuclear element‐1 (LINE‐1) reverse transcriptase and associated innate immune activation. Phase II findings showed exploratory signals for respiratory and ALSFRS‐R decline, as well as changes in neurofilament levels and inflammatory mediators [362, 363].

Neuroprotective strategies increasingly target mitochondrial energy metabolism, cellular stress adaptation, and neuronal survival signaling. CNM‐Au8 and pridopidine represent these approaches, but neither met the primary efficacy endpoint in HEALEY ALS Platform Trial analyses [364, 365]. The Phase III PREVAiLS trial is evaluating pridopidine in ALS, reflecting continued investigation of this neuroprotective strategy [366].

Cell therapy also remains under investigation in ALS, particularly MSC‐based strategies with potential neurotrophic and immunomodulatory effects. NurOwn and lenzumestrocel are two representative approaches in this field. NurOwn is an autologous MSC therapy based on neurotrophic factor‐secreting cells (MSC‐NTF). A randomized, placebo‐controlled Phase III trial did not meet its primary endpoint, although subgroup and biomarker analyses suggested possible effects in selected patients [367]. In 2025, the United States Food and Drug Administration (US FDA) cleared a new Phase IIIb trial of NurOwn [368]. A 2023 real‐world study of lenzumestrocel suggested an association with longer survival [369]. Although the Phase III ALSUMMIT trial did not meet its primary endpoint overall, a 2025 analysis reported a possible benefit in patients with slower disease progression [370].

Emerging ALS therapies have advanced in molecular stratification, targeted mechanisms, and platform trial design, but major evidentiary and translational limitations remain. The key efficacy challenge is to translate mechanistic plausibility into confirmed clinical benefit. As shown by CNM‐Au8 and pridopidine, drugs with neuroprotective or stress‐modulating rationales may still fail to meet clinical efficacy endpoints. Biological signals, biomarker changes, and exploratory subgroup trends therefore require validation in larger confirmatory trials [352].

Safety and the balance of risks and benefits are also major considerations. Gene‐based therapies pose challenges related to CNS delivery, repeated or long‐term administration, durability of target suppression, and off‐target effects. These therapies raise concerns about adverse effects, dose tolerability, and interference with normal cellular defenses. For cell‐based approaches, manufacturing consistency, delivery routes, long‐term monitoring, and immune‐related risks require further attention. Evaluation must therefore extend beyond short‐term tolerability and exploratory efficacy signals [371].

Real‐world implementation barriers also remain substantial. Genotype‐ or pathway‐specific therapies narrow the eligible population. Many candidates also require biomarker screening, repeated intrathecal administration, long‐term follow‐up, or specialized centers. Given the progressive and fatal course of ALS, early positive signals must be interpreted cautiously so that expectations do not exceed the evidence.

### 5.4 Huntington's Disease

#### 5.4.1 Established Therapeutic Strategies

At present, no approved disease‐modifying therapy is available for HD. Established treatment therefore centers on symptom control, preservation of function and multidisciplinary supportive care.

Among motor symptoms, chorea has the strongest evidence base for pharmacological treatment. Vesicular monoamine transporter 2 (VMAT2) inhibitors are the main pharmacological options. Tetrabenazine reduces chorea scores but requires individualized dose titration and monitoring for depression, suicidal ideation, parkinsonism, and dysphagia [372, 373]. Deutetrabenazine and valbenazine have also reduced HD‐related chorea in randomized trials and are established treatment options [374, 375].

VMAT2 inhibitors are central to chorea management, but many patients also require individualized treatment for psychiatric and behavioral symptoms. When irritability, aggression, psychosis, or sleep disturbance coexist with chorea, antipsychotics may help manage excessive movements and behavioral symptoms. However, the evidence supporting their use is weaker than that for VMAT2 inhibitors [376]. Depression, anxiety, apathy, insomnia, and other neuropsychiatric symptoms should be managed with pharmacological and psychosocial interventions tailored to each patient's presentation rather than a uniform regimen [377].

Multidisciplinary supportive care is essential to established HD treatment. Physiotherapy, occupational therapy, speech and swallowing management, nutritional assessment, social support, and advance care planning should be integrated throughout the disease course [378]. Clinical practice recommendations support aerobic exercise, alone or combined with resistance training, to improve fitness and motor function. They also support supervised gait training to improve gait and individualized balance exercises to improve balance [379, 380]. Early swallowing assessment, diet texture modification, and nutritional monitoring help manage dysphagia and weight loss and reduce the risks of aspiration, dehydration, and malnutrition [381].

#### 5.4.2 Emerging Therapeutic Strategies

Unlike most established interventions, which primarily aim to relieve symptoms, emerging HD therapies increasingly aim to modify disease progression. The main strategies are to reduce *HTT* expression or inhibit somatic CAG repeat expansion at an earlier pathogenic step. Both aim to intervene before toxic protein accumulation and neuronal vulnerability intensify.

Direct reduction of *HTT* expression remains the most clinically advanced emerging therapeutic strategy. The earliest clinical evidence of target engagement came from the nonallele‐specific ASO tominersen. A Phase I/IIa study showed dose‐dependent reductions in CSF mutant HTT, indicating that central *HTT* lowering is feasible in humans [382]. Dosing in GENERATION HD1, which enrolled individuals with manifest HD, was discontinued after an unfavorable benefit–risk assessment [383]. GENERATION HD2 tested lower exposure in people with prodromal or early manifest HD, but it did not meet its efficacy objective. Roche therefore discontinued further clinical development of tominersen in July 2026 [384]. These outcomes underscore the need to distinguish target engagement from clinical benefit when designing future *HTT*‐lowering approaches and selecting patients and doses.

In parallel, preclinical studies have advanced *HTT* lowering toward allele‐specific approaches. Iwamoto et al. developed the stereopure ASO WVE‐003 to target the single‐nucleotide polymorphism (SNP) rs362273, designated SNP3 in that study, when it is linked to the expanded *HTT* allele. By controlling phosphorothioate backbone stereochemistry and introducing phosphoryl guanidine modifications, WVE‐003 favors ribonuclease H (RNase H)‐mediated cleavage of mutant *HTT* transcripts. In patient‐derived neurons and HD mouse models, it produced potent and durable mutant HTT lowering while relatively preserving wild‐type HTT [385]. Compared with nonselective *HTT*‐lowering controls, WVE‐003 maintained potency while showing greater allele selectivity and durability. Compared with earlier SNP‐targeted candidates that did not consistently lower CSF mutant HTT, WVE‐003 appears to have a more favorable activity profile. A related commentary highlighted WVE‐003 as an example of the shift toward selectively lowering mutant HTT while potentially preserving the neuroprotective functions of wild‐type HTT. This approach may guide future patient stratification, molecular design, and precision therapy [386].

Single‐administration intracerebral gene therapy offers another approach. AMT‐130 uses AAV‐mediated microRNA delivery and is designed to produce sustained *HTT* suppression in the striatum. uniQure's AMT‐130 studies are registered on ClinicalTrials.gov under NCT04120493 [387] and NCT05243017 [388]. In 2025, company‐reported 36‐month topline data suggested slower decline on the composite UHDRS (cUHDRS) among high‐dose recipients than among externally matched controls [389]. Spark and Roche also initiated a randomized, sham‐controlled Phase I/II trial of SPK‐10001 (RG6662) in 2025 [390]. Sustained AAV‐mediated *HTT* lowering is therefore being evaluated in more than one clinical program.

Development also extends to oral agents and next‐generation RNA‐lowering approaches. Votoplam (PTC518) is an oral splicing modulator. An early study in healthy volunteers showed that votoplam reduced systemic HTT mRNA and HTT protein levels through splicing modulation [391]. In 2025, PIVOT‐HD met its primary blood HTT‐lowering endpoint and reported sustained HTT lowering, together with preliminary biomarker and clinical signals [392]. ALN‐HTT02, an RNA interference therapy targeting exon 1 of *HTT*, has entered Phase I development [393].

An important recent shift in HD therapeutic research is the recognition of somatic CAG repeat expansion as an upstream target. A 2025 *Cell* study linked extensive somatic CAG repeat expansion to striatal neuronal degeneration [95]. Longitudinal data further associated peripheral blood measures of somatic expansion with subsequent caudate and putaminal atrophy [213]. Together, these findings support targeting pathogenic repeat expansion earlier in disease progression, alongside efforts to reduce toxic protein exposure. DNA repair pathways therefore offer potential therapeutic targets. A 2025 study found that an *MSH3*‐targeting ASO reduced *MSH3* expression and prevented further CAG repeat expansion in patient‐derived striatal neurons [394]. In vivo CRISPR screening also identified modifier genes that regulate somatic expansion [96].

SKY‐0515 is among the most clinically advanced candidates pursuing this strategy. This oral RNA splicing modulator is designed to lower mutant HTT and *PMS1* expression. Phase I results showed reduced blood *HTT* mRNA levels [395]. The randomized, double‐blind, placebo‐controlled Phase II/III FALCON‐HD trial has also begun [396]. This approach aims to address both toxic protein production and a driver of repeat expansion. Its long‐term efficacy and appropriate patient population remain to be established in prospective studies.

Although emerging HD therapies increasingly target disease mechanisms, no disease‐modifying therapy has yet been approved. A major limitation of *HTT*‐lowering strategies is that target engagement does not establish a safe and effective therapeutic window. Tominersen reduced CSF mutant HTT, yet neither GENERATION HD1 nor GENERATION HD2 demonstrated clinical benefit [383, 384]. Dose, dosing interval, patient stratification and timing of intervention therefore remain critical. Nonallele‐specific *HTT* lowering also raises long‐term safety concerns because wild‐type HTT has important physiological functions, while the consequences of excessive suppression remain incompletely defined [397].

Delivery also presents important safety and practical challenges. AAV‐based therapies such as AMT‐130 require MRI‐guided stereotactic neurosurgery and cannot be readily reversed after administration. Public trial materials report that AMT‐130 lowers both wild‐type and mutant HTT in animal studies. They also note that its long‐term effects in adults remain unknown and that recipients may become ineligible for future gene therapies [398].

The maturity of clinical evidence remains a limitation across these approaches. Preclinical WVE‐003 studies showed selective lowering of mutant HTT while preserving wild‐type HTT, but its applicability is limited to eligible SNP3 carriers [385]. Other candidates also rely heavily on early studies, interim analyses, or company‐released data rather than mature evidence from prospective controlled trials.

Strategies targeting somatic expansion and DNA repair pathways address upstream disease mechanisms but remain largely preclinical. Evidence linking extensive somatic CAG repeat expansion to degeneration of striatal medium spiny neurons strengthens the rationale for this approach [95]. Long‐term administration, tissue‐specific effects, and the safety window under sustained treatment still require clinical evaluation.

### 5.5 Frontotemporal Dementia

The therapeutic challenge in FTD arises from its clinicopathological heterogeneity. No US FDA‐approved disease‐modifying therapy is currently available for FTD. Care therefore focuses on supportive interventions and individualized management of behavioral, affective, language, and motor symptoms.

#### 5.5.1 Established Therapeutic Strategies

Established FTD treatment cannot reverse neurodegeneration. Its goals are to reduce behavioral dysregulation, maintain function and ease caregiving burden. Because impaired insight, poor social judgement, stereotyped behaviors, and safety risks are common, nonpharmacological interventions remain foundational. These include environmental structuring, simplified routines, caregiver education, language training, occupational therapy, and physiotherapy. For PPA, communication strategy training and speech‐language therapy are particularly important. For prominent behavioral symptoms, stabilizing the care environment and reducing triggers may improve daily functioning more effectively than adding medication [399, 400, 401, 402, 403].

Serotonergic agents are among the better‐supported pharmacological options for behavioral symptoms in FTD. Trazodone and some selective serotonin reuptake inhibitors (SSRIs) may improve disinhibition, irritability, stereotyped, or compulsive‐like behaviors and eating abnormalities. They have not shown a clear benefit for cognition. Trazodone reduced Neuropsychiatric Inventory scores in an early randomized trial [404]. Later studies and meta‐analyses suggest modest SSRI benefits for selected behavioral symptoms, with no clear cognitive benefit [405, 406].

Cognitive‐enhancing treatments adapted from AD have yielded largely disappointing results in FTD. Memantine has not shown a clear clinical benefit and may have unfavorable effects on some outcomes. Cholinesterase inhibitors also lack convincing evidence of benefit and may worsen impulsivity or disinhibition in patients with prominent behavioral symptoms [407, 408].

In patients with severe aggression, agitation or psychotic symptoms that pose safety risks, antipsychotics may be used cautiously for short‐term symptom control. Their use addresses immediate risks rather than the underlying disease and requires monitoring for sedation and extrapyramidal adverse effects [105].

#### 5.5.2 Emerging Therapeutic Strategies

Emerging FTD trials increasingly use genotype, protein pathology, and molecular mechanism to guide patient selection. These include restoring progranulin in *GRN*‐associated FTD, reducing toxic RNA and DRPs in *C9orf72*‐associated FTD, and targeting tau through antibodies, expression‐lowering agents, and related approaches [409, 410, 411].

Progranulin restoration has reached the most advanced stage of clinical testing among these approaches. Latozinemab blocks sortilin‐mediated progranulin degradation and is a representative antibody in this group. A 2024 Phase I study reported acceptable safety and increased plasma and CSF progranulin levels, supporting target engagement [412]. In 2025, the company reported topline results from the 96‐week Phase III INFRONT‐3 trial. Latozinemab met the plasma progranulin biomarker coprimary endpoint but did not meet the clinical coprimary endpoint of slowing progression in *GRN*‐associated FTD [413].

Unlike monoclonal antibody approaches that increase progranulin levels, *GRN* gene replacement aims to directly correct the pathogenic protein deficiency. An interim analysis of the Phase I/II open‐label PR006 trial, reported in 2024, showed that one‐time cisterna magna administration was generally acceptably safe, and that CSF progranulin increased in all participants, indicating clear target bioactivity. However, transient increases in NfL also suggest the need for continued attention to dorsal root ganglia toxicity, while the rate of clinical progression remained within the broad natural‐history range previously observed in FTD. Therefore, efficacy still requires validation through longer follow‐up and larger cohorts [409].

For *C9orf72*‐associated FTD, therapeutic efforts are directed toward suppressing repeat‐expanded transcripts and their downstream toxic products. ASO programs such as WVE‐004 are being developed within this framework. Existing preclinical studies indicate that these agents can relatively selectively reduce repeat‐containing abnormal transcripts [410], while early clinical results from FOCUS‐C9 released by the company also suggest target engagement and preliminary safety signals [414]. However, as with progranulin‐directed strategies, whether target engagement can translate into clinically meaningful slowing of disease progression remains insufficiently proven. For tau‐directed therapies, research strategies likewise increasingly emphasize pathology‐enriched populations, such as early‐stage trials in primary tauopathy groups including *MAPT*‐associated disease, nonfluent variant PPA, PSP, and corticobasal syndrome [411].

Notably, emerging therapeutic strategies are not entirely synonymous with disease‐modifying therapy. Trials targeting symptoms through newer mechanistic approaches also warrant attention. For example, a 2025 study on intranasal oxytocin suggested that intermittent administration may produce modest improvement in apathy in patients with FTD [415]. A Phase IIa study of rotigotine also suggested that dopaminergic modulation may improve selected cognitive functions related to frontal networks. However, these findings remain early exploratory signals and are not yet sufficient to alter the current reality that established therapy is grounded in behavioral management and caregiving support [416]. Rather, these studies indicate that therapeutic innovation in FTD is occurring not only at the level of genes and protein pathology, but also at the more refined level of symptom‐related neural circuits.

Emerging FTD therapies have not yet consistently translated into clear clinical benefit. PR006 illustrates this challenge: it requires invasive cisterna magna administration and was associated with immune and inflammatory safety signals, including anti‐AAV9 antibodies, CSF pleocytosis, transient NfL increases, and one case of hearing loss [409]. Therefore, progranulin gene restoration is most plausibly favorable in genetically confirmed *GRN*‐associated FTD with defined molecular deficiency, relatively early disease, and a remaining therapeutic window, rather than in unstratified broader FTD cohorts.

The *C9orf72*‐related ASO approach highlights a broader problem: target engagement does not automatically translate into disease modification. WVE‐004 can selectively reduce repeat‐expanded transcripts and DRPs while largely preserving normal C9orf72 protein expression, but peer‐reviewed evidence of clinical benefit in human FTD remains limited [105, 410, 417]. Tau‐directed strategies face a similar dilemma: despite the central role of tau in FTLD‐tau, no tau‐directed disease‐modifying therapy has been approved, partly because clinical syndromes often do not reliably predict underlying tau pathology and late‐stage or mixed pathology may dilute efficacy signals [418].

Symptom‐targeted emerging strategies appear to be safer but have limited efficacy. Intranasal oxytocin produced only modest improvement in apathy and was generally well tolerated [415], while rotigotine showed mostly mild adverse effects but failed to demonstrate convincing clinical efficacy in bvFTD [416].

### 5.6 Friedreich's Ataxia

Therapeutic development for FRDA has centered on FXN deficiency and its subsequent effects on downstream mitochondrial dysfunction, OS and disrupted iron homeostasis [305].

#### 5.6.1 Established Therapeutic Strategies

Established FRDA care combines comprehensive supportive management with approved disease‐modifying pharmacotherapy. FRDA affects the nervous system and heart and can impair glucose metabolism, mobility, speech, and swallowing. Standard care therefore requires coordinated input from neurology, cardiology, rehabilitation, nutrition, and speech and swallowing services. This multidisciplinary approach addresses cardiomyopathy, arrhythmias, glucose metabolism abnormalities, gait instability, dysarthria, and dysphagia [109].

Omaveloxolone is the first approved disease‐modifying therapy for FRDA and is indicated for patients aged 16 years or older. Its precise mechanism remains incompletely defined. Available evidence suggests that its neuroprotective effects involve Nrf2‐related responses, redox regulation, and attenuation of mitochondrial stress [419].

In MOXIe Part 2, omaveloxolone improved scores on the modified Friedreich Ataxia Rating Scale (mFARS) relative to placebo at 48 weeks [420]. Analyses of the open‐label extension (OLE), including propensity score‐matched comparisons with the Friedreich Ataxia Clinical Outcome Measures Study (FACOMS), suggested slower progression over approximately 3 years [421]. The observed benefit is best interpreted as slower deterioration, without evidence that established disease is reversed. The clinical meaningfulness of the mFARS improvement remains debated. Improvements in upper‐limb function, mobility and fall‐related outcomes have not been consistently demonstrated. Long‐term evidence relies mainly on OLE data and comparisons with matched natural‐history cohorts. Without continued randomized control, the magnitude, durability, and real‐world generalizability of benefit remain uncertain [422].

#### 5.6.2 Emerging Therapeutic Strategies

Compared with established treatments, emerging FRDA therapies more closely target processes in the disease cascade. Four main approaches are under investigation: downstream metabolic and OS modulation, FXN protein replacement, gene addition, and transcriptional or epigenetic reactivation of the *FXN* gene.

Vatiquinone and leriglitazone illustrate a common challenge for downstream modulatory therapies in FRDA: strong mechanistic rationale has yet to yield confirmed clinical benefit. Long‐term follow‐up studies have suggested potential benefit from vatiquinone [423]. However, the pivotal MOVE‐FA trial did not meet its primary endpoint. Vatiquinone therefore cannot yet be regarded as an established disease‐modifying therapy [424].

Leriglitazone faces a similar evidence gap. The FRAMES study did not meet its primary imaging endpoint, but showed a trend toward less iron accumulation in the dentate nucleus and favorable results on several exploratory measures. These findings suggest possible target engagement or tissue‐level biological effects. These signals do not establish consistent, reproducible and clinically meaningful functional improvement. Peripheral oedema also raises questions about leriglitazone's long‐term clinical acceptability [425].

Nomlabofusp takes a more direct approach by replacing deficient FXN protein. Early clinical studies have reported an acceptable short‐term safety profile, characterized its pharmacokinetics, and shown preliminary pharmacodynamic activity, including increased FXN levels in peripheral tissues. Whether nomlabofusp provides sustained clinical benefit remains to be determined, particularly through longer‐term open‐label extension studies [426].

Gene addition aims to compensate for the underlying *FXN* gene defect, although delivery, safety, and access remain challenges. LX2006 primarily targets FRDA‐associated cardiomyopathy and remains under clinical evaluation. Its long‐term efficacy, the reproducibility of cardiac benefit and its long‐term safety profile remain to be established [427].

SGT‐212 uses a more complex delivery strategy: infusion into the dentate nuclei followed by intravenous administration. Current evaluation focuses mainly on safety and tolerability. Although this approach has therapeutic potential, it also poses procedural and immunological challenges and requires specialist centers and long‐term follow‐up [428].

Transcriptional or epigenetic reactivation of *FXN* remains a complementary strategy. High‐dose nicotinamide studies suggest that FXN levels can be increased, providing proof of concept for pharmacological modulation of *FXN* silencing. Clinical disease modification remains unproven [429].

### 5.7 Multiple Sclerosis

Compared with most NDDs discussed above, MS has a relatively mature therapeutic landscape. Numerous approved disease‐modifying therapies (DMTs) have altered the natural history of relapsing MS. However, therapeutic priorities have shifted beyond the suppression of peripheral inflammation. Major challenges now include controlling compartmentalized CNS inflammation, slowing progression independent of relapse activity (PIRA), promoting remyelination and preserving neurological function.

#### 5.7.1 Established Therapeutic Strategies

Established MS treatment requires DMT selection tailored to disease subtype, disease activity, and individual risk tolerance. For relapsing MS, DMT options include injectable agents, oral immunomodulators, cell‐trafficking inhibitors, anti‐CD20 monoclonal antibodies, and immune reconstitution therapies. These treatments reduce relapses and MRI lesion activity. However, their effects on long‐term disability accumulation, particularly PIRA, remain limited [430, 431, 432].

Earlier platform therapies, including interferon beta and glatiramer acetate, remain useful in patients with low‐to‐moderate disease activity. Teriflunomide and fumarates provide further options when tolerability, convenience, or safety is a priority. Selected sphingosine‐1‐phosphate receptor modulators can also be considered according to disease activity and individual risk. Clinical practice increasingly considers high‐efficacy therapies earlier in the disease course, including natalizumab and anti‐CD20 therapies. Natalizumab reduces relapse rates and the risk of sustained disability progression. Its use requires assessment of progressive multifocal leukoencephalopathy risk, including stratification by John Cunningham virus antibody status [433, 434].

B‐cell‐depleting therapies now play a central role in high‐efficacy MS treatment. Ocrelizumab reduces disease activity in relapsing MS and slows disability progression in primary progressive MS (PPMS). Compared with teriflunomide, ofatumumab reduces relapses and MRI lesion activity [435, 436, 437].

Established treatment options for progressive MS remain fewer than those for relapsing MS. Siponimod reduces the risk of disability progression in active secondary progressive MS (SPMS), making it an important option for this subgroup [438]. Cladribine is an oral immune reconstitution therapy for relapsing forms of MS. Given its safety profile, it is generally considered after an inadequate response to, or intolerance of, another MS therapy [439].

#### 5.7.2 Emerging Therapeutic Strategies

Emerging therapeutic strategies for MS can currently be broadly grouped into three main directions: deeper modulation of CNS inflammation, true remyelination and neural repair, and cell‐based therapies aimed at stronger immune resetting or tissue support.

Among these strategies, Bruton's tyrosine kinase (BTK) inhibitors are the closest to potentially changing clinical practice in the near term, because they aim to extend the high‐efficacy anti‐inflammatory approach while also offering the possibility of CNS penetration and modulation of microglia and compartmentalized inflammation. However, the clinical development of BTK inhibitors has not followed a uniformly successful path. For instance, evobrutinib failed to show superiority over teriflunomide in two Phase III trials. Similarly, tolebrutinib did not demonstrate superiority over teriflunomide in relapsing MS, but it reduced the risk of disability progression in nonrelapsing SPMS [440, 441, 442].

Fenebrutinib represents one of the more translationally promising candidates within the BTK inhibitor class. The Phase II FENopta study showed that fenebrutinib had acceptable tolerability and significantly and durably suppressed the formation of new focal inflammatory brain lesions in relapsing MS. Based on these findings, fenebrutinib is regarded as a candidate worthy of continued attention. However, based on currently published evidence, its clinical value still lies mainly in early anti‐inflammatory activity and pharmacological feasibility, whereas its true benefit for long‐term disability progression, especially in progressive disease biology, requires further validation [443, 444].

If BTK inhibitors aim to deepen inflammatory control, remyelination strategies aim to address the tissue repair problem that established DMTs have not yet truly solved. In the ReBUILD trial, clemastine provided human proof of concept for remyelination by shortening visual evoked potential P100 latency, a milestone result that has long been repeatedly cited in the field. However, subsequent evidence quickly reminded the field that remyelination cannot be directly extrapolated into long‐term clinical benefit simply because biomarker changes are observed. Studies published in 2025 further suggested that, in case of progressive MS, clemastine may instead accelerate disability accumulation by enhancing pyroptosis [445, 446].

Cell therapy represents the most resetting‐ or repair‐oriented end of emerging MS therapeutics. Autologous hematopoietic stem cell transplantation (AHSCT) is no longer merely a conceptual approach. A 2019 randomized clinical trial showed that, in highly active relapsing‐remitting MS (RRMS), nonmyeloablative AHSCT significantly prolonged time to disease progression compared with continued DMTs. By 2025, a *Nature Reviews Neurology* consensus statement had positioned AHSCT as an acceptable option for aggressive RRMS after failure of high‐efficacy DMTs. Nevertheless, the toxicity of AHSCT, its dependence on center expertise and its strict patient‐selection requirements mean that it is unlikely to become a universally applicable strategy. By contrast, cell therapies for progressive MS, such as MSC‐derived neural progenitors (MSC‐NP), have entered randomized, placebo‐controlled trials, but they remain at an early stage overall and are still far from becoming standard treatment [447, 448, 449]. Therefore, the most realistic near‐term role of cell therapy in MS is not to replace existing DMTs, but to provide stronger immune resetting or neural repair potential for a small subset of patients with high risk or substantial unmet need.

Based on current evidence, BTK inhibitors are closest to near‐term translation, but questions regarding safety and patient selection remain unresolved. Remyelination is theoretically the most promising direction, but remains limited by endpoint design and mechanistic heterogeneity. Cell therapy may produce larger effect sizes, but its accessibility and treatment burden mean that it is likely to serve only selected subgroups in the near term (Figure 4).

**FIGURE 4:**
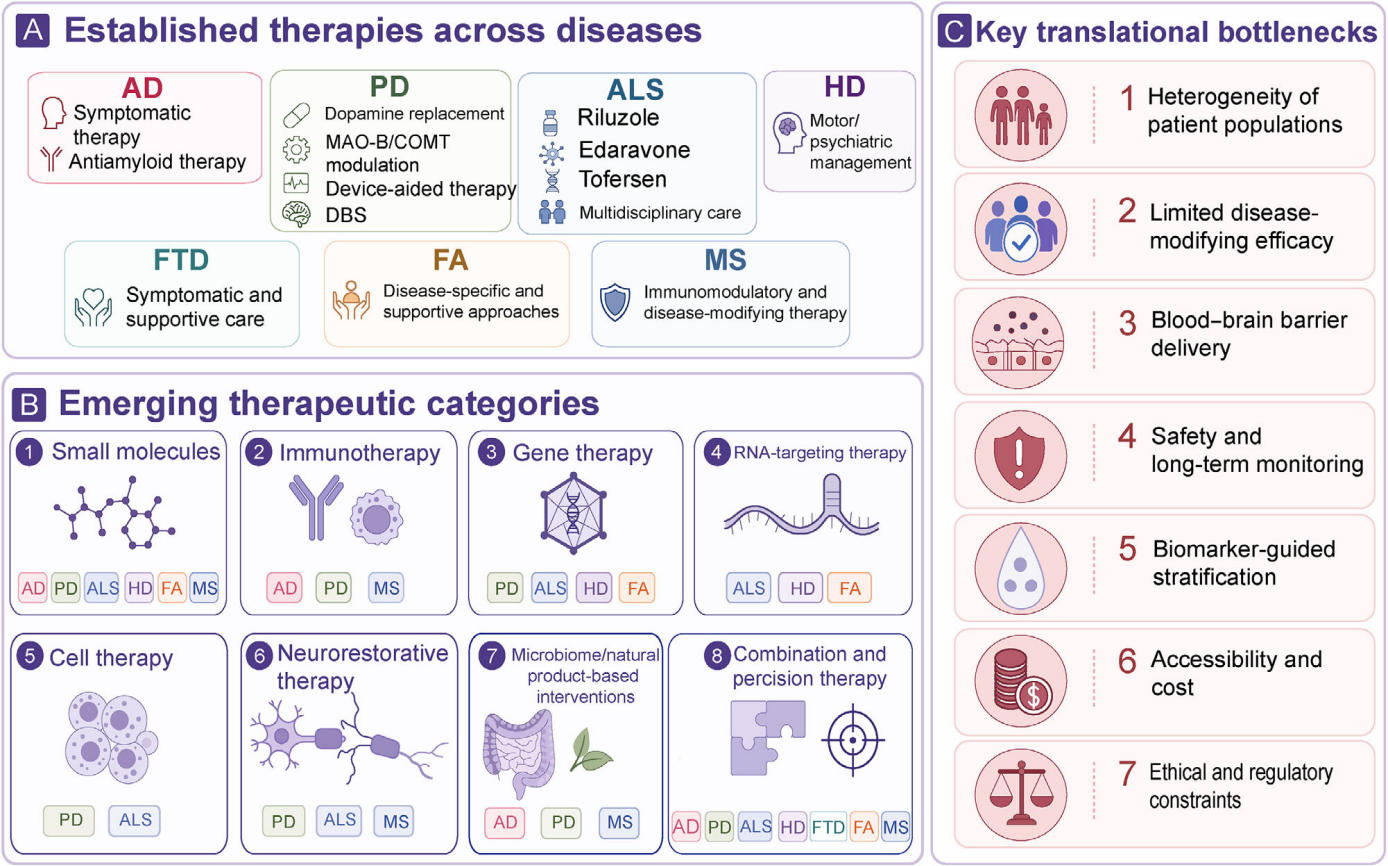
Established and emerging therapeutic landscape of NDDs. (A) Current management strategies vary across NDDs but remain centered on symptomatic control, supportive care, and selected disease‐modifying approaches where available. (B) Emerging therapeutic paradigms increasingly target upstream disease mechanisms and include small molecules, immunotherapy, gene therapy, RNA‐targeting approaches, cell‐based strategies, neurorestorative interventions, and systems‐level therapeutic concepts. (C) Translation into durable clinical benefit remains limited by disease heterogeneity, late diagnosis, inadequate patient stratification, delivery barriers, long‐term safety concerns, trial complexity, and unequal access. Future progress will depend on earlier intervention, biomarker‐guided precision medicine, combination therapy, and more equitable real‐world implementation. *Abbreviations*: AD, Alzheimer's disease; ALS, amyotrophic lateral sclerosis; COMT, catechol‐O‐methyltransferase; DBS, deep brain stimulation; FA, Friedreich's ataxia; FTD, frontotemporal dementia; HD, Huntington's disease; MAO‐B, monoamine oxidase B; MS, multiple sclerosis; NDDs, neurodegenerative diseases; PD, Parkinson's disease.

### 5.8 Key Preclinical and Clinical Evidence Landscape

The preceding overview shows that NDDs therapeutic development is shifting from symptomatic treatment toward mechanism‐directed approaches, including pathological protein clearance, nucleic acid and gene regulation, mitochondrial and OS repair, immunoinflammatory modulation, cell replacement, and neural repair. Evidence maturity varies substantially: anti‐Aβ monoclonal antibodies in AD, tofersen in *SOD1*‐ALS, omaveloxolone in FRDA, and multiple DMTs in MS have entered clinical practice, whereas many other strategies remain in dose exploration, safety validation, surrogate endpoint assessment, or subtype‐specific verification. Importantly, preclinical or biomarker improvement does not necessarily translate into stable and reproducible functional benefit for patients (Table 1).

**TABLE 1:** Representative preclinical and clinical evidence for mechanism‐based therapies in neurodegenerative diseases.

Pathway/mechanism	Disease examples	Representative agents or strategies	Preclinical rationale	Clinical evidence stage	Trial registration number(s)	Key findings	Main limitations and safety concerns	References
Aβ clearance and amyloid pathology modification	AD	Lecanemab; donanemab	Aβ aggregation is considered an important early event in the AD pathological cascade. Anti‐Aβ antibodies reduce amyloid burden in animal models, early human studies, and biomarker‐confirmed clinical cohorts.	Phase III/approved in selected regions	Lecanemab: Clarity AD, NCT03887455; donanemab: TRAILBLAZER‐ALZ 2, NCT04437511	Lecanemab and donanemab reduce amyloid burden and slow cognitive and functional decline in early symptomatic AD.	Clinical benefit is modest and depends on early patient selection and amyloid confirmation. ARIA‐E, ARIA‐H, infusion reactions, and anticoagulant or thrombolytic‐related risks require close monitoring.	[450, 451]
GLP‐1 receptor‐related metabolic and neuroinflammatory modulation	PD	GLP‐1 receptor agonists, including lixisenatide and exenatide	GLP‐1 receptor agonists show metabolic, anti‐inflammatory, and potentially neuroprotective effects in toxin‐induced and inflammation‐related PD models.	Phase II to Phase 3/mixed evidence	Lixisenatide: LixiPark, NCT03439943; exenatide: Exenatide‐PD3, NCT04232969	Lixisenatide was associated with less progression of motor disability in early PD, whereas later Phase IIIevidence with exenatide did not confirm superiority over placebo.	Class‐level disease‐modifying efficacy remains uncertain. Gastrointestinal adverse effects may limit tolerability.	[343]
Dopaminergic cell replacement and circuit restoration	PD	Stem‐cell‐derived dopaminergic progenitors, including hESC‐ and iPSC‐derived products	Stem‐cell‐derived dopaminergic progenitors can survive, differentiate, and restore striatal dopaminergic signaling in experimental models.	Phase I/early‐phase clinical trials	Bemdaneprocel/BRT‐DA01: exPDite, NCT04802733	Early clinical studies suggest feasibility, acceptable short‐term safety, and potential motor benefit signals.	Evidence is limited by small samples and early‐stage designs. Long‐term monitoring is needed for graft survival, ectopic growth, immunosuppression, tumor risk, and surgical complications.	[347]
Gene‐specific RNA lowering	ALS; HD; FUS‐ALS; C9orf72‐related ALS/FTD	Tofersen; tominersen; ulefnersen/jacifusen; WVE‐004	ASOs can reduce pathogenic messenger RNA or toxic protein expression, thereby targeting upstream mechanisms in genetically defined disease subtypes.	Approved for SOD1‐ALS; mixed, negative, or early evidence in HD and rare ALS/FTD genetic subtypes	Tofersen: VALOR, NCT02623699; OLE, NCT03070119; tominersen: GENERATION HD1, NCT03761849; ulefnersen/jacifusen: FUSION, NCT04768972; WVE‐004: FOCUS‐C9, NCT04931862; OLE, NCT05683860	Tofersen lowers CSF SOD1 and plasma NfL and received accelerated approval. Tominersen showed that target lowering does not automatically translate into clinical benefit. Jacifusen and WVE‐004 illustrate next‐generation genotype‐specific approaches.	Applicable populations are narrow. Intrathecal administration and long‐term target suppression are burdensome, and some programs have required discontinuation, refinement or further validation.	[383, 452, 453, 454]
Mitochondrial and oxidative stress modulation	FRDA	Omaveloxolone	Frataxin deficiency causes mitochondrial dysfunction, oxidative stress, and impaired iron–sulfur cluster biogenesis. Nrf2 activation may improve antioxidant and mitochondrial homeostasis.	Phase II/approved in selected regions	MOXIe, NCT02255435	The MOXIe trial showed improvement in mFARS scores, supporting omaveloxolone as the first approved therapy for FRDA.	Use is limited to specific patient groups. Liver enzymes and lipid parameters require monitoring. Established structural neurological damage is unlikely to be reversed.	[420]
Progranulin restoration and lysosomal correction	FTD‐GRN	Progranulin‐raising/restoration strategies, including latozinemab and PR006/AAV‐GRN gene therapy	GRN haploinsufficiency causes lysosomal dysfunction, lipofuscin accumulation, and neuroinflammation. Progranulin replacement improves disease‐relevant phenotypes in cellular and animal models.	Phase Ⅰ/ IIto Phase III	Latozinemab: INFRONT‐2, NCT03987295; INFRONT‐3, NCT04374136; PR006/AAV‐GRN: PROCLAIM, NCT04408625	PR006 early data suggest increased CSF progranulin and improved lysosomal biomarkers. Latozinemab increases progranulin levels.	Clinical outcome evidence remains limited. FTD‐GRN cohorts are small and heterogeneous. AAV therapy requires careful monitoring for immune responses, dorsal root ganglion toxicity, and long‐term safety.	[409, 455]
Remyelination and myelin repair	MS	Clemastine; other remyelination candidates	Promoting oligodendrocyte precursor cell differentiation and myelin repair could theoretically improve chronic demyelinating injury.	Phase II/mixed evidence	Clemastine: ReBUILD, NCT02040298; TRAP‐MS clemastine arm, NCT03109288	The ReBUILD trial suggested improvement in visual evoked potential latency in chronic demyelinating optic neuropathy.	Subsequent progressive MS work raised concerns regarding safety and disease progression. Links between repair‐oriented endpoints and functional improvement remain unstable.	[445, 446]
Immune reset and compartmentalized inflammation control	MS	AHSCT; BTK inhibitors; MSC‐NP	AHSCT resets peripheral immunity. BTK inhibitors target B‐cell and myeloid‐cell signaling. MSC‐NP cells may provide immunomodulatory and repair‐supportive effects.	Phase II to Phase III	Autologous hematopoietic stem cell transplantation: MIST, NCT00273364; tolebrutinib: HERCULES, NCT04411641; fenebrutinib: FENopta, NCT05119569; MSC‐NP cells: NCT03355365	AHSCT shows strong effects in highly active RRMS. Tolebrutinib reduced disability progression in nonrelapsing SPMS. Fenebrutinib suppressed MRI inflammatory activity.	AHSCT has substantial toxicity and depends on center expertise. BTK inhibitors require long‐term safety validation. MSC‐NP requires larger trials and standardized protocols.	[442, 443, 447, 449]

Abbreviations: AAV, adeno‐associated virus; AD, Alzheimer's disease; AHSCT, autologous hematopoietic stem cell transplantation; ALS, amyotrophic lateral sclerosis; Aβ, amyloid‐β; ARIA, amyloid‐related imaging abnormalities; ASO, antisense oligonucleotide; BTK, Bruton tyrosine kinase; CSF, cerebrospinal fluid; FRDA, Friedreich ataxia; FTD, frontotemporal dementia; FUS, fused in sarcoma; GLP‐1, glucagon‐like peptide‐1; GRN, granulin precursor; HD, Huntington's disease; hESC, human embryonic stem cell; iPSC, induced pluripotent stem cell; mFARS, modified Friedreich Ataxia Rating Scale; MRI, magnetic resonance imaging; MS, multiple sclerosis; MSC‐NP, mesenchymal stem cell‐derived neural progenitor; NCT, ClinicalTrials.gov identifier; NfL, neurofilament light chain; Nrf2, nuclear factor erythroid 2‐related factor 2; OLE, open‐label extension; PD, Parkinson's disease; RRMS, relapsing‐remitting multiple sclerosis; SPMS, secondary progressive multiple sclerosis.

Mechanistically, therapies with more mature evidence usually share three common features: well‐defined pathological or genetic targets, biomarkers for patient selection and pharmacodynamic monitoring, and endpoints sensitive enough to capture treatment effects. Anti‐Aβ antibodies rely on amyloid biomarkers, tofersen on SOD1 stratification and NfL changes, omaveloxolone on mitochondrial OS biology, and high‐efficacy MS immunotherapies on mature MRI, relapse, and disability endpoints. By contrast, strategies targeting tau, α‐syn, TDP‐43, HTT lowering, remyelination, and cell replacement remain constrained by target timing, delivery, long‐term safety, endpoint sensitivity, and disease heterogeneity (Table 2).

**TABLE 2:** Comparative summary of representative therapeutic agents across neurodegenerative diseases.

Disease	Agent/strategy	Mechanism of action	Development stage	Trial registration number(s)	Key trial outcome	Safety profile	Evidence interpretation	References
AD	Lecanemab	Anti‐Aβ protofibril monoclonal antibody	Phase III; approved in selected regions	Clarity AD, NCT03887455	Clarity AD showed reduced amyloid burden and slower cognitive and functional decline over 18 months.	ARIA‐E, ARIA‐H, and infusion reactions; APOE ε4 carriers have higher ARIA risk.	Marks the shift of AD therapy toward biomarker‐guided disease modification, although effect size and eligible population remain limited.	[450]
AD	Donanemab	Anti‐Aβ plaque monoclonal antibody	Phase III; approved in selected regions	TRAILBLAZER‐ALZ 2, NCT04437511	TRAILBLAZER‐ALZ 2 showed slower clinical progression in early symptomatic AD.	ARIA‐E, ARIA‐H, and infusion reactions; MRI monitoring is required.	Supports clinical validity of amyloid clearance while reinforcing the need for safety monitoring and precise patient selection.	[451]
PD	Lixisenatide	GLP‐1 receptor agonist; metabolic and inflammatory modulation	Phase II	LixiPark, NCT03439943	Motor disability worsened less over 12 months than in the placebo group.	Nausea, vomiting, and other gastrointestinal adverse effects are common.	Provides early human evidence for a metabolic‐neuroprotective strategy but does not replace standard dopaminergic treatment.	[343]
PD	Bemdaneprocel and related stem‐cell‐derived dopaminergic progenitors	Cell replacement of lost dopaminergic neurons	Phase Ⅰ/early clinical development	exPDite, NCT04802733	Early studies suggest feasibility, acceptable safety, and potential motor benefit signals.	Surgical risk, immunosuppression, long‐term graft behavior, and tumor risk require monitoring.	Represents a major regenerative medicine direction for PD, but current evidence is mainly safety and feasibility evidence.	[347]
ALS	Tofersen	ASO targeting SOD1 messenger RNA	Accelerated approval for SOD1‐ALS	VALOR, NCT02623699; OLE, NCT03070119	Lowers CSF SOD1 and plasma NfL. Primary clinical endpoint was not significantly improved, but extension data support possible benefit of earlier treatment.	Intrathecal administration‐related risks; CSF leukocyte and protein elevation, pain, and fatigue.	A milestone for genotype‐driven ALS therapy and a key example of the regulatory value and limitations of surrogate endpoints.	[452]
HD	Tominersen	ASO lowering HTT transcripts	Phase III stopped; further clinical development discontinued in July 2026	GENERATION HD1, NCT03761849	Early studies showed mutant HTT lowering, but GENERATION HD1 was stopped early.	High‐dose or frequent dosing may produce an unfavorable benefit–risk relationship.	Shows that HTT lowering is mechanistically plausible, but therapeutic window, dose, and patient selection determine success.	[383, 384]
HD	AMT‐130	AAV5‐miHTT gene therapy	Early‐phase clinical development	US study, NCT04120493; European study, NCT05243017	Early data suggest potential slowing of disease progression, but evidence remains exploratory.	Neurosurgical delivery risks and long‐term safety of gene expression require evaluation.	Represents one‐time gene therapy for HD, but controlled studies and regulatory confirmation are still needed.	[456]
FTD‐GRN	Latozinemab	Antisortilin monoclonal antibody increasing progranulin levels	Phase II/ III	INFRONT‐2, NCT03987295; INFRONT‐3, NCT04374136	Increases progranulin levels; clinical benefit remains uncertain.	Infusion‐related risks; long‐term safety and clinical efficacy require confirmation.	Reflects the move from syndrome‐based FTD treatment to genotype‐specific therapy, but biomarker correction does not equal proven clinical benefit.	[455]
FTD‐GRN	PR006/AAV‐GRN	AAV‐mediated GRN gene replacement	Phase Ⅰ/ II	PROCLAIM, NCT04408625	Early data show increased CSF progranulin and improved lysosomal biomarkers.	CSF pleocytosis, immune responses, and long‐term AAV safety require monitoring.	An important genetic correction approach for FTD‐GRN, but evidence remains early‐stage.	[409]
FRDA	Omaveloxolone	Nrf2 activation; mitochondrial and oxidative stress modulation	Phase II; approved in selected regions	MOXIe, NCT02255435	MOXIe showed improvement in mFARS, supporting approval as the first FRDA therapy.	Liver enzymes and lipid parameters require monitoring.	A landmark FRDA breakthrough, but it should be viewed as functional slowing rather than curative therapy.	[420]
MS	Clemastine	Putative remyelination through OPC differentiation	Phase II; mixed evidence	ReBUILD, NCT02040298; TRAP‐MS clemastine arm, NCT03109288	ReBUILD showed improvement in visual evoked potential latency.	Somnolence and fatigue; later progressive MS work raised disability‐worsening concerns.	Shows that remyelination can be detected in humans, but repair endpoints and safety remain challenging.	[445, 446]
MS	Tolebrutinib/fenebrutinib	BTK inhibition targeting B‐cell and myeloid‐cell signaling	Phase II/ III	Tolebrutinib: HERCULES, NCT04411641; fenebrutinib: FENopta, NCT05119569	Tolebrutinib reduced disability progression in nonrelapsing SPMS. Fenebrutinib suppressed MRI inflammatory activity.	Liver toxicity, infection, and long‐term immune modulation risks require attention.	BTK inhibitors may connect peripheral immunity and CNS compartmentalized inflammation, but efficacy differs across MS subtypes.	[442, 443]
MS	AHSCT	Immune ablation and immune reconstitution	Randomized clinical evidence; selected clinical use	MIST, NCT00273364	Superior to continued DMT in highly active RRMS and delayed disease progression.	Infection, infertility, treatment‐related toxicity, and center‐expertise dependence.	Appropriate for selected highly active treatment‐refractory RRMS patients, not a universal MS strategy.	[447]
MS	MSC‐NP	Intrathecal cell therapy with immunomodulatory and repair potential	Phase II	NCT03355365	Preliminary signals of neurological benefit in progressive MS.	Administration procedure, cell‐manufacturing standardization, and long‐term safety require confirmation.	A repair‐oriented strategy for progressive MS, but far from standardized clinical application.	[449]

*Abbreviations*: AAV, adeno‐associated virus; AD, Alzheimer's disease; AHSCT, autologous hematopoietic stem cell transplantation; ALS, amyotrophic lateral sclerosis; Aβ, Amyloid‐β; APOE, apolipoprotein E; ARIA, amyloid‐related imaging abnormalities; ASO, antisense oligonucleotide; ARIA‐E, amyloid‐related imaging abnormalities with edema or effusion; ARIA‐H, amyloid‐related imaging abnormalities with hemosiderin deposition; BTK, Bruton tyrosine kinase; CNS, central nervous system; CSF, cerebrospinal fluid; DMT, disease‐modifying therapy; FRDA, Friedreich ataxia; FTD, frontotemporal dementia; GLP‐1, glucagon‐like peptide‐1; GRN, granulin precursor; HD, Huntington's disease; HTT, huntingtin; mFARS, modified Friedreich Ataxia Rating Scale; miHTT, microRNA targeting huntingtin; MRI, magnetic resonance imaging; MS, multiple sclerosis; MSC‐NP, mesenchymal stem cell‐derived neural progenitor; NCT, ClinicalTrials.gov identifier; NfL, neurofilament light chain; Nrf2, nuclear factor erythroid 2‐related factor 2; OLE, open‐label extension; OPC, oligodendrocyte precursor cell; PD, Parkinson's disease; RRMS, relapsing‐remitting multiple sclerosis; SPMS, secondary progressive multiple sclerosis.

## 6 Conclusion and Prospects

Future therapeutic strategies for NDDs are moving beyond single‐target interventions toward systemic modulation, mechanistic synergy, and disease modification. This chapter summarizes emerging paradigms represented by gut microbiome modulation, fungal‐derived bioactive compounds, and plant‐derived natural products, which remain exploratory but reflect a shift from pathogenic protein‐centered models toward systemic homeostatic regulation. Then, this chapter outlines future priorities, including biomarker‐based stratification, longitudinal cohorts, platform trial design, and equity and accessibility in translational medicine.

### 6.1 Emerging Paradigms in Neurodegenerative Therapy

Therapeutic research in NDDs is shifting from symptom‐focused and single‐target approaches toward systemic modulation, multipathway interventions, and modification of disease progression. Conventional pharmacotherapy remains valuable, but achieving durable effects across NDDs on shared pathological processes remains difficult. These processes include protein aggregation, neuroinflammation, OS, mitochondrial dysfunction, gut–brain axis dysregulation, and neural network degeneration [457].

Gut microbiota modulation has attracted substantial attention as a systemic therapeutic approach. The microbiota can influence host metabolism, immune regulation, barrier homeostasis, and CNS function through microbial metabolites, including short‐chain fatty acids and neurotransmitter‐like molecules, as well as peripheral immune responses and vagal signaling. The microbiota–gut–brain axis is associated with neuroinflammation, microglial activation, intestinal barrier dysfunction, and protein aggregation in AD, PD, ALS, and MS [457]. Probiotics, postbiotics, dietary interventions, fecal microbiota transplantation, and metabolite‐targeted strategies may therefore complement conventional pharmacotherapy. In PD, gastrointestinal symptoms and peripheral inflammatory changes may precede motor symptoms, suggesting a potential opportunity for early intervention and risk modulation [458]. However, evidence still comes largely from animal studies, observational research and small clinical studies. Causality, optimal intervention windows and doses, long‐term safety and patient stratification remain unclear.

Fungal‐derived bioactive compounds and mycotherapy are also being investigated as multitarget approaches. Medicinal fungi contain polysaccharides, triterpenoids, erinacines, and hericenones. These compounds may have antioxidant and anti‐inflammatory effects and may regulate autophagy, mitochondrial function, and neurotrophic signaling. Specifically, bioactive compounds from *Ganoderma lucidum* may influence Aβ, tau, neuroinflammation, and OS. Components of *Hericium erinaceus* have been associated with neurotrophic support, synaptic plasticity, and cognitive improvement [459, 460]. However, clinical translation of mycotherapy is constrained by complex composition, nonstandardized extraction, variable active compound content, limited pharmacokinetic evidence, and small clinical trials. Future studies should identify active components, clarify mechanisms, and establish optimal doses and clinical endpoints.

Plant‐derived natural products and phytotherapy represent another multitarget approach. Curcumin, *Ginkgo biloba* extract, resveratrol, polyphenols, flavonoids, and terpenoids may affect OS, neuroinflammation, protein aggregation, mitochondrial injury, apoptosis, and synaptic function. These potential effects align with the multifactorial pathology of NDDs [461, 462]. However, clinical translation of many candidates is hindered by low bioavailability, limited BBB penetration, formulation variability, uncertain dosing, and heterogeneous clinical study designs [463]. Future phytotherapy research should prioritize structural optimization, improved delivery, combination strategies, and high‐quality randomized controlled trials over the identification of additional natural products.

Overall, gut microbiota modulation, fungal‐derived bioactive compounds, and plant‐derived natural products illustrate the shift from single‐target interventions toward systemic regulation in NDD therapy.

### 6.2 Future Directions

NDDs are highly heterogeneous and have complex pathological mechanisms that vary by disease stage. Future therapeutic strategies should therefore move beyond uniform treatment models and match interventions to disease mechanisms, biological subtypes and disease stages. In the near term, research should prioritize defining patient subgroups by genetic profiles, biomarker signatures, pathological features, and patterns of clinical progression. For example, *APOE* ε4 status or polygenic risk scores may help enrich AD trials, while *LRRK2* or *GBA1* mutation status may guide patient selection in PD. In MS and other inflammation‐associated NDDs, inflammatory signatures, including microglial PET signals and cytokine profiles, may further support patient stratification [464].

Developing robust biomarker systems is another central research priority. Imaging, fluid, and digital biomarkers are essential for early diagnosis, disease monitoring, assessment of target engagement, and evaluation of therapeutic response. Potential components include amyloid PET, tau PET, investigational α‐syn PET, neuroinflammation imaging, and advanced MRI measures. Plasma or CSF NfL, glial fibrillary acidic protein, neurogranin, cytokine panels, and digital cognitive or motor measures may add complementary information. Together, these biomarkers may provide a more sensitive framework for evaluating disease progression and treatment efficacy [465, 466]. Future clinical trials should integrate these markers with clinical outcomes in composite endpoints. This may improve the sensitivity of therapeutic evaluation, particularly in slowly progressive diseases.

Clinical trial design must also evolve alongside precision medicine. Adaptive, platform, umbrella, and basket trial designs may allow multiple agents, combinations, and disease subgroups to be tested more efficiently within shared frameworks. Such designs may reduce sample‐size requirements and trial duration while supporting interim go/no‐go decisions based on biomarker and clinical readouts [467]. Enrichment strategies should enroll biologically defined participants, such as amyloid‐positive individuals in AD, *LRRK2* or *GBA1* mutation carriers in PD, and *SOD1* or *C9orf72* subgroups in ALS. This approach may improve trial power and better align therapeutic mechanisms with patient biology.

Rational development of multimodal and combination therapies is another important direction. NDDs involve interacting mechanisms, including protein misfolding, impaired proteostasis, mitochondrial dysfunction, OS, neuroinflammation, synaptic failure, axonal injury, and glial dysfunction. Single‐target interventions may therefore be insufficient for many patients. Future strategies could combine antiamyloid and antitau agents in AD, pair immunomodulation with neuroprotective or neurotrophic strategies, or integrate anti‐inflammatory and proteostasis‐enhancing interventions [464]. Combination therapies require strong preclinical evidence of additive or synergistic effects, careful dose optimization, and rigorous safety monitoring to limit cumulative toxicity.

Translational validation remains a major bottleneck in NDD research [468]. To reduce clinical failures after preclinical success, future studies should use models that better reflect human disease biology. These include aged and multihit animal models, human iPSC‐derived cells, organoids, and disease‐in‐a‐dish platforms. Before large clinical trials, candidate therapies should demonstrate pharmacokinetic feasibility, BBB penetration, CNS distribution, and target engagement. Phase 0 microdosing studies, PET occupancy studies, CSF sampling, and early pharmacodynamic readouts may help establish whether a therapy reaches its intended target before costly late‐phase trials begin.

Advanced delivery technologies will also be critical for the next generation of NDD therapies. Improved viral vectors, engineered AAV capsids, ASOs, lipid nanoparticles for CNS delivery, intranasal formulations, BBB‐penetrant small molecules, and intrathecal or intracerebroventricular approaches may expand therapeutic possibilities. These approaches also require standardized delivery protocols, long‐term safety monitoring, and reliable biomarkers of distribution and target engagement. For gene and cell therapies, immune responses, off‐target effects, tumorigenicity, and neuroinflammation require long‐term surveillance, while manufacturing consistency must be ensured.

Several therapeutic areas merit particular attention. In MS, BTK inhibitors may help target chronic compartmentalized inflammation, particularly in progressive disease. Remyelination remains an important therapeutic goal, but agents targeting LINGO1 and repurposed drugs such as clemastine require further efficacy and safety evaluation. Studies should use rigorous imaging and functional endpoints, including myelin water fraction MRI, myelin PET ligands, visual evoked potentials, and gait measures [445, 469]. Cell‐based therapies, including AHSCT, MSC‐derived approaches, and iPSC‐derived neural cells, may be especially relevant to severe or refractory disease. Their clinical application requires rigorous potency assays, standardized manufacturing, and long‐term follow‐up.

Systemic and multitarget interventions, including gut microbiome modulation, fungal‐derived bioactive compounds, and plant‐derived natural products, are also emerging directions in NDD therapy. Gut–brain axis interventions, such as probiotics, prebiotics, fecal microbiota transplantation, and microbial metabolite modulation, may be relevant to PD, AD, and MS. Gut pathology and immune–metabolic changes may arise early in these diseases [470]. Fungal‐ and plant‐derived compounds, including *Hericium* and *Ganoderma* constituents, curcumin, *Ginkgo* extract, resveratrol, polyphenols, flavonoids, and terpenoids, may have multitarget neuroprotective effects. Proposed actions include modulation of OS, neuroinflammation, protein aggregation, mitochondrial dysfunction, apoptosis, and synaptic impairment [471, 472]. Their clinical translation requires chemical standardization, improved bioavailability, pharmacokinetic optimization, and rigorously designed randomized controlled trials.

Overall, the development of future NDD therapies should be iterative, biomarker guided, and mechanism oriented. Near‐term priorities include validating stratification markers, establishing patient registries, and launching small adaptive trials with integrated surrogate endpoints. Mid‐term efforts should focus on large platform trials, rational combinations, digital biomarkers, and regulatory frameworks for complex interventions. Long‐term goals include refining personalized treatment algorithms, demonstrating meaningful functional benefits and implementing effective therapies in clinical practice. Progress in this field will depend on therapeutic innovation, data sharing, specialized trial networks, manufacturing capacity, regulatory coordination, equitable access, and multidisciplinary collaboration. Together, these efforts may shift NDD therapy from symptom control toward mechanism‐based, stage‐specific, and personalized disease modification.

## Author Contributions

ZMH wrote the original draft and finished the table and the figure; JJZ and GQW reviewed and edited the manuscript; YXL finished the figure, and FZ revised and checked the article. All authors have read and approved the final manuscript.

## Funding

This study was supported by the National Natural Science Foundation of China (No. 82460711) and the Science and Technology Foundation of Guizhou Province (Nos. ZYD[2025]022, ZD[2026]163, and KJLYRC‐[2026]038).

## Ethics Statement

The authors have nothing to report.

## Conflicts of Interest

The authors declare no conflicts of interest.

## Data Availability

No new data were generated or analyzed in support of this review. All data and findings discussed in this paper are derived from previously published studies, which have been properly cited in the reference list.
